# RNA-Seq Analysis of *Quercus pubescens* Leaves: *De Novo* Transcriptome Assembly, Annotation and Functional Markers Development

**DOI:** 10.1371/journal.pone.0112487

**Published:** 2014-11-13

**Authors:** Sara Torre, Massimiliano Tattini, Cecilia Brunetti, Silvia Fineschi, Alessio Fini, Francesco Ferrini, Federico Sebastiani

**Affiliations:** 1 Institute for Plant Protection, Department of Biology, Agricultural and Food Sciences, The National Research Council of Italy (CNR), Sesto Fiorentino, Italy; 2 Department of Agri-Food and Environmental Sciences, University of Florence, Sesto Fiorentino, Italy; 3 Institute for Biosciences and BioResources, Department of Biology, Agricultural and Food Sciences, The National Research Council of Italy (CNR), Sesto Fiorentino, Italy; The National Orchid Conservation Center of China; The Orchid Conservation & Research Center of Shenzhen, China

## Abstract

*Quercus pubescens* Willd., a species distributed from Spain to southwest Asia, ranks high for drought tolerance among European oaks. *Q. pubescens* performs a role of outstanding significance in most Mediterranean forest ecosystems, but few mechanistic studies have been conducted to explore its response to environmental constrains, due to the lack of genomic resources. In our study, we performed a deep transcriptomic sequencing in *Q. pubescens* leaves, including de novo assembly, functional annotation and the identification of new molecular markers. Our results are a pre-requisite for undertaking molecular functional studies, and may give support in population and association genetic studies.

254,265,700 clean reads were generated by the Illumina HiSeq 2000 platform, with an average length of 98 bp. De novo assembly, using CLC Genomics, produced 96,006 contigs, having a mean length of 618 bp. Sequence similarity analyses against seven public databases (Uniprot, NR, RefSeq and KOGs at NCBI, Pfam, InterPro and KEGG) resulted in 83,065 transcripts annotated with gene descriptions, conserved protein domains, or gene ontology terms. These annotations and local BLAST allowed identify genes specifically associated with mechanisms of drought avoidance. Finally, 14,202 microsatellite markers and 18,425 single nucleotide polymorphisms (SNPs) were, *in silico*, discovered in assembled and annotated sequences. We completed a successful global analysis of the *Q. pubescens* leaf transcriptome using RNA-seq. The assembled and annotated sequences together with newly discovered molecular markers provide genomic information for functional genomic studies in *Q. pubescens*, with special emphasis to response mechanisms to severe constrain of the Mediterranean climate. Our tools enable comparative genomics studies on other *Quercus* species taking advantage of large intra-specific ecophysiological differences.

## Introduction

The genus *Quercus* (oaks), a member of the Fagaceae family, comprises ∼400 deciduous and evergreen trees and shrubs distributed from tropical to boreal regions, thus constituting significant components of forests in the northern hemisphere [1]. According to their widespread distribution, the genus differentiated into numerous species and populations, with different morphological and physiological traits, enabling their occurrence in areas with contrasting climates. *Quercus* species have been classified as drought-resistant [2], thus allowing their distribution in a wide range of soils [3]. In Central Europe, *Quercus robur* L., *Q. petraea* [Matt.] Libel. and *Q. pubescens* Willd. are most abundant white oak species, and inhabits areas with contrasting temperature and precipitation [4]. *Quercus pubescens* Willd. (pubescent or downy oak) is a thermophilous tree native to southern Europe, widely distributed from Spain to southwest Asia and from European Mediterranean coastlines to central Europe. It is a xerophilous species and typically grows on dry, lime-rich soils in the sub-Mediterranean region, which is characterised by hot dry summers and mild dry winters [5].

Global climate change imposes to Mediterranean regions a general increase of temperatures and aridity, coupled with a higher frequency of extreme climatic events such as heat waves and late-winter frosts [6], [7]. This represents a complex challenge for plants inhabiting Mediterranean-type ecosystems [8], in turn greatly affecting the composition, structure and functioning of forest ecosystems and, hence, the productivity of natural and managed forests [9]–[11]. Therefore, successful forest management practices in the future have to take into account a conversion from forests currently dominated by relatively drought-sensitive coniferous species (i.e., Norway spruce and Scots pine) and European beech (*Fagus sylvatica* L.) [12], [13] to an admix of more drought- and temperature-tolerant trees, such as oak (*Quercus* spp.) [9], [14].

Oaks are generally considered to be resistant to drought because of deep-penetrating roots, xeromorphic leaves and very effective control of stomata opening (thus reducing transpiration water loss) [2], [15]. Nonetheless, drought tolerance may differ considerably among oak species and provenances, reflecting adaptation to environments with different water availability [16]. Therefore, the characterization of adaptive genetic variation is an issue of outstanding significance for managing natural resources (and gene conservation) as well as to predict their ability to cope with constrains imposed by the global climate change [17]–[19].

Recent advances in high-throughput sequencing technologies offer novel opportunities in functional genomics, as well as in discovering genes and developing molecular markers in non-model plants [20]. The massively parallel sequencing of RNA (RNA-Seq) represents a powerful tool for transcription profiling, providing a rapid access to a collection of expressed sequences (transcriptome), as compared with traditional expressed sequence tag (EST) sequencing. RNA-Seq technology has been successfully applied in different domains of life, ranging from animals to yeast up to a wide array of model and non-model plants [21]–[27].

A large number of sequenced transcripts, with approximately 2.5 million of ESTs deposited in databases, has been reported for Fagaceae family (*Quercus*, *Castanea*, *Fagus*, and *Castanopsis*, Fagaceae Genomics Web: http://www.fagaceae.org/) [28]. On the other hand, genomic resources for *Q. pubescens* are very limited at the present (no ESTs, just 178 nt sequences, mainly chloroplast fragments). Therefore, development of genomic resources for *Q. pubescens* is a timely issue to support molecular biology studies at different levels of scale (from evolutionary ecology to comparative and functional genomics).

In our study, we used Illumina RNA-seq technology to analyse the transcriptome of *Q. pubescens* leaves. We generated 254,265,700 clean reads containing a total of about 25 Gb of sequence data. *De novo* assembly was then applied followed by gene annotation and functional classification. Our RNA-seq analysis generated the first *Q. pubescens* consensus transcriptome and provides an unlimited set of molecular markers.

## Material and Methods

### Plant materials and RNA isolation

Juvenile and mature *Q. pubescens* leaves from 4 half-sib 4-years old plants were collected. These plants were cultivated in 3L pots and grew up in the greenhouse, under natural daylight condition. Five chloroplast (cp) and four nuclear (nu) microsatellites (SSRs) were used to test for parentage analysis (data not shown). Partially and fully expanded leaves at 2–6 internodes from apex were collected and immediately frozen in liquid nitrogen and stored at −80°C until processing. Seven cDNA libraries (one from each sampling) were prepared using pooled mRNA. Total RNA of each sample was extracted separately from 100 mg of leaves using Qiagen RNeasy Plant Mini Kit (Qiagen, Valencia, CA) following manufacturer's instructions with minor modifications: 10% v/v of N-lauroyl sarcosine 20% w/v was added to RLC buffer for each sample followed by incubation at 70°C for 10 min with vigorous shaking before proceeding with the standard protocol. The RNA samples were treated with 2 units of DNase I (Ambion, Life Technologies, Gaithersburg, MD) for 30 min at 37°C to remove contaminating genomic DNA.

The quality of each RNA sample was checked by means of agarose gel electrophoresis and quantified with Qubit fluorometer (Life Technologies). RNA integrity was confirmed using the Agilent 2100 Bioanalyzer (Agilent Technologies, Santa Clara, CA, USA), with a minimum RNA integrated number value of 7.5. Isolated RNAs from different samples were dissolved in RNase-free water and stored in −80°C freezer until subsequent analysis.

### Transcriptome sequencing and assembly

Work flow of the present study is reported in the flow chart in Figure S1. The RNA samples were pooled equally to construct the cDNA libraries and processed as outlined in Illumina's “TruSeq RNA-seq Sample Prep kit” (Illumina, Inc., CA, USA).

The sample was loaded on Illumina flow cell and sequenced at ultra-high throughput on Illumina HiSeq2000 (Illumina Inc.). After sequencing of the cDNA library, base calling using Illumina pipeline software was used to transform the raw image data generated into sequence information. The CLC Genomics Workbench version 6.5.1 (CLC-Bio, Aarhus, Denmark) was selected for de novo transcriptome assembly in the present study because the CLC software has a faster computing pace with comparable or better assembly results than other bioinformatics programs [29]. The Illumina reads were trimmed at the ends by quality scores on the basis of the presence of ambiguous nucleotides (typically N) (maximum value set at 2) using a modified version of the Mott algorithm (the quality limit was set to 0.05), as implemented on CLC Genomics Workbench. Then, high-quality reads with overlaps were assembled to generate contigs using default parameters, obtaining 96006 contigs. RNA sequencing was performed at IGA Technology Services Srl Service Provider (Udine, Italy).

The raw reads produced in this study have been deposited at the Sequence Read Archive (SRA) of the National Centre of Biotechnology Information under the accession number SRP043444.

### Functional Annotation and classification

All the transcripts were compared with the sequences of various databases to extract the maximum possible information based on sequence and functional similarity. For assignments of predicted gene descriptions, the BLASTX algorithm was used to search for homologous sequences (e-value cut off ≤1.0e-10) against the Viridiplantae, the UniProt (Swiss-Prot and TrEMBL) and NCBI RefSeq (collection of comprehensive, integrated, non-redundant, well-annotated set of proteins) protein databases. Based on the BLAST hits identified, GO (Gene Ontology, the Gene Ontology Consortium, 2000; www.geneontology.org) annotation (biological process, molecular functions and cellular components terms) was performed using BLAST2GO [30]: in particular, GO terms associated with each BLAST hit were retrieved (mapping step) and GO annotation assignment (annotation step) to the query sequences was carried out using the following annotation score parameters; e-value hit filter (default  = 1.0e-6), annotation cut-off (default  = 55), GO-weight (default  = 5), hsp-hit coverage cut off (default  = 0). Within the same routine an enzyme classification number (EC number) was assigned using a combination of similarity searches and statistical analysis terms.

In addition, conserved domains/motifs were further identified using Inter-ProScan, an on-line sequence search plug-in within the BLAST2GO program that combines different protein signature recognition methods, with the InterPro database (version 30.0,) and the resulting GO terms were merged with the GO term results from the annotation step of Blast2GO.

The Kyoto Encyclopedia of Genes and Genomes (KEGG) database is used extensively to reveal molecular interaction network and metabolic pathways [31]. KEGG pathways annotation was performed by mapping the sequences obtained from BLAST2GO to the contents of the KEGG metabolic pathway database.

Domain-based comparisons with KOG (EuKaryotic Orthologous Groups, a eukaryote-specific version of the Clusters of Orthologous Groups (COG) tool for identifying ortholog and paralog proteins) and Pfam (version 27.0) databases were performed using RPS-BLAST (Reverse PSI-BLAST) tool from locally installed NCBI BLAST+ v2.2.18 software.

### Identification of putative candidate genes involved in drought avoidance

To uncover the potential candidate genes related to drought stress in *Q. pubescens*, 35 genes involved in epidermal development such as stomata, cuticle waxes, trichomes and root hairs [32] in *Arabidopsis* were selected to screen the potential orthologs from the contig dataset by Local BLASTN with E-value cutoff of 1e-5 (ftp://ftp.ncbi.nlm.nih.gov/blast/executables/LATEST-BLAST/).

### RT-PCR validation of transcripts

Total RNA from *Q. pubescens* leaves was reverse-transcribed by using SuperScript III Reverse Transcriptase (Invitrogen) and oligo(dT)18. Twenty-one annotated transcripts were selected for RT-PCR validation. Forward (Fwd) and reverse (Rev) primers were designed using Primer3. The fragments were cloned in pGEM-T easy vector (Promega, Madison, WI) and transformed into JM109 cells. All recombinant plasmids were sequenced with the M13 universal primers using the Big Dye Terminator v.3.1 Cycle Sequencing Kit (Applied Biosystems, Foster City, CA) and run on an ABI 3730 DNA Analyzer (Applied Biosystems, Foster City, CA) automatic sequencer. The plasmids with inserts longer than 1000 bp were treated with GPS-1 Genome Priming System (NEB, Beverly, MA) to generate and sequence random subclones.

### SSR and SNP identification and validation

Mining of the simple sequence repeats (SSRs) present in the contigs of *Q. pubescens* was performed using MIcroSAtellite identification tool (MISA, http://pgrc.ipk-gatersleben.de/misa/), considering di-, tri-, tetra-, penta- and hexa-nucleotide motifs with a minimum of 5 contiguous repeat units [33]. The PRIMER3 software [34] was used to design forward and reverse primers flanking the SSR containing sequence [33]. For validation of SSR primers, total DNA was extracted from young leaves of eight *Q. pubescens* seedlings from two Italian populations (four for each one). For DNA extraction, the Dneasy Plant mini kit (Qiagen, Valencia, CA) was used following the manifacturer's instructions, and the amplification reactions were carried out as reported in Sebastiani et al. [35].

All the contigs from the transcriptome were used to mine SNPs and InDel markers. These markers were detected by the alignment of individual reads against contigs from the assembly using CLC Genomics Workbench ver. 6.5.1 that is based on the Neighborhood Quality Standard (NQS) algorithm of Altshuler et al. [36]. A minimum of two individual reads aligning with the references need to show the variant alleles in order to consider a sequence difference as a true polymorphism. From all variations, the high-confidence variations were screened based on the parameters that three or more non-duplicate reads confirm the same variations in both forward and reverse reads and single nucleotide-InDels should be found in at least 10% of total unique sequencing reads.

In order to validate the *in silico* SNPs from the transcripts assembly, 9 fragments carrying ten putative SNPs were amplified in the four genotypes used for the transcriptome assembly. Newly designed primer pairs and fragment sizes are listed in Figure S2. The amplified fragments were cloned into pGEM-T easy vector and transformed into JM109 cells. From two to eight randomly chosen clones for each genotype were sequenced in the forward direction with the M13 universal primer using the Big Dye Terminator v.3.1 Cycle Sequencing Kit (Applied Biosystems, Foster City, CA) and run on an ABI 3730 DNA Analyzer (Applied Biosystems) automatic sequencer.

## Results and Discussion

### Transcriptome Sequencing output and de novo Assembly

High-throughput sequencing technology has been extensively used to explore the transcriptome profile of non-model plant species. Our study depicts, for the first time, the fully expressed genome information of the downy-oak leaf. We performed RNA-Seq of leaf bulk sample of four different individuals to unveil *Q. pubescens* leaf transcriptome. The Illumina sequencing procedure generated 310,521,410 raw nucleotide paired reads 100 bp long. Removal of adapter sequences, ambiguous and low-quality reads, resulted in 254,265,700 clean reads of average read length 98.2 bp, corresponding to a complete dataset of about 25 Gb of sequence data (Table 1).

**Table 1:** Summary of RNA-Seq and *de novo* sequence assembly for *Q. pubescens*.

Total raw reads	310,521,410
Total clean reads	254,265,700
Number of contigs	96,006
Mean lenght of contigs (bp)	618
N75	401
N50	910
N25	1,889

N75 length is defined as the length N for which 75% of all bases in the sequences are in a sequence of length L <N.

N50 length is defined as the length N for which 50% of all bases in the sequences are in a sequence of length L <N.

N25 length is defined as the length N for which 25% of all bases in the sequences are in a sequence of length L <N.

*De-novo* assembly of high quality reads was performed through CLC Genomics Workbench, and produced 96,006 contigs, thus creating a foundational reference transcriptome for *Q. pubescens* leaf expressed genome. N50 and N75 (parameters widely used for assessing the sequence quality) were 910 bp and 401 bp, respectively. Both indexes are higher than those previously reported for a wide range of plant transcriptome assemblies [25], [37]–[39]. The mean length of these assembled contigs is 618 bp and the sequence length ranges from 129 bp to more than 10,000 bp. These values are comparable to those found in other non-model plants, which indeed range from 200 bp to 750 bp [24], [32], [40]. Our results are of particular interest, as transcriptome analysis was performed just on leaf tissue, and confirm the suitability of mRNA-Seq for extensive and accurate assembly of non-model plant transcriptomes.

### Transcript Annotation

Functional annotation of novel plant transcriptome is great challenge for non-model plants for which references genome/gene sequences in databases are scarce. Here, annotation of the *Q. pubescens* transcriptome sequences was based on two levels of sequence similarity, namely sequence-based and domain-based alignments. Sequence-based alignments were performed against four public databases, including the NCBI non-redundant protein (Nr) database, RefSeq protein (NP) at NCBI, UniProt (Swiss-Prot and TrEMBL), and the Kyoto Encyclopedia of Genes and Genomes (KEGG) using BLASTX algorithm with a significant E-value threshold of 1e^−5^. Domain/family searches contained Hidden Markov Model (HMM) domain/family searches in both the InterPro and Pfam databases and BLASTX alignments against the EuKaryotic Orthologous Groups (KOG) database at NCBI [25]. The E-value threshold was also set at ≤1e^−5^. Annotations statistics of BLASTX hits and domain hits are summarized in Table 2.

**Table 2:** Summary of annotations of assembled *Q. pubescens* contigs.

Database	Number of trascripts annotated	Percentage of trascripts annotated
Viridiplantae Nr	68,285	71.12%
RefSeq	77,623	80.85%
UniProt	73,148	76.19%
InterPro	48,622	50.27%
KOG	19,146	19.94%
Pfam	20,448	21.29%
GO	8,536	12.54%
KEGG	4,050	4.22%

**Figure 1:**
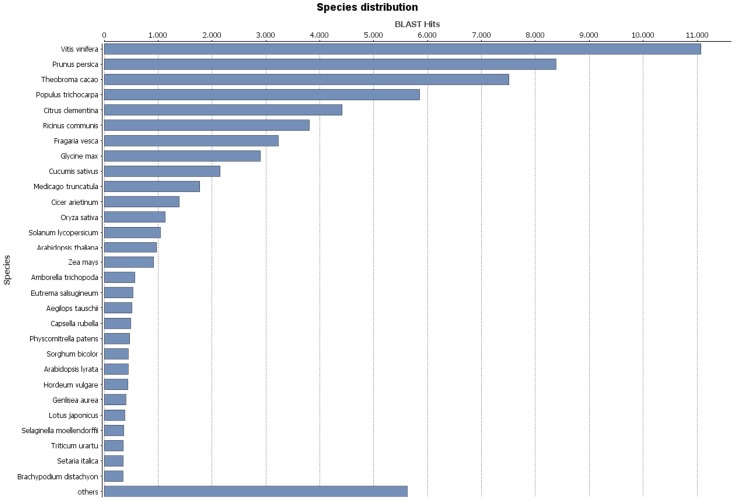
Hit species distribution of BLASTX matches of *Q. pubescens* contigs. Proportion of *Q. pubescens* contigs with similarity to sequences from Nr Viridiplantae protein database.

All assembled sequences were first aligned to the 2,935,608 protein sequences from a custom-made NCBI non-redundant database (Nr) filtered to give only Viridiplantae proteic sequences, which returned 68,285 significant BLAST hits (71.1%, see Table 2 and Table S1). The mapping rates of the *Quercus* contigs against the NP RefSeq and UniProt databases were 80.8% and 76.2%, respectively (Table 2 and significant hits in Table S1). Importantly, the high mapping rates of the contigs to the proteic databases suggest that most of the contigs can be translated into proteins. BLASTX hits and top hits in terms of the total hit numbers to all transcripts were mostly observed with *Vitis vinifera* (11,074 hits), *Prunus persica* (8,382 hits), *Theobroma cacao* (7,512 hits), *Populus trichocarpa* (5,853 hits) and *Citrus clementine* (4,416 hits) (Figure 1). Although *V. vinifera* shows highest correlation with *Q. pubescens*, only 10% of annotated transcripts in *Q. pubescens* finds homology with protein sequences in *V. vinifera* (Figure 1). Indeed *Quercus* and *Vitis* belong to different orders, i.e. Fagales (Fagaceae,) and Vitales (Vitaceae), respectively.

### Conserved domain annotation

**Figure 2:**
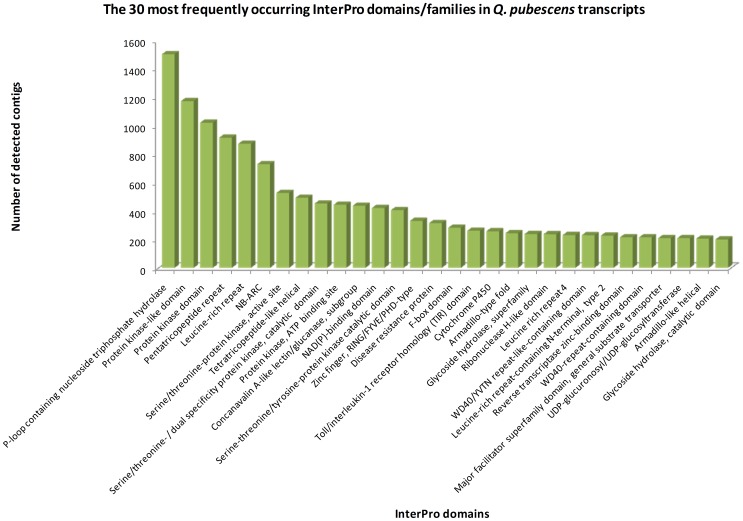
Histogram of the 30 most abundant InterPro domains revealed by the InterProScan annotation of the high quality *Quercus pubescens* transcript set.

To further exploit the potential function of the transcriptome sequences conserved domains in *Q. pubescens* were identified against the InterPro, Pfam and KOG databases. The aim of the annotation against this database is to identify similarity at domain level, where proteins have little similarity at sequence level but may share conserved structural domains. Searches against the InterPro database revealed 48266 top hits categorized into 5550 domain/families (Table 2). InterPro domains/families were therefore sorted according to the number of *Q. pubescens* contigs contained in each InterPro domain. The 30 most abundant InterPro domains/families have been reported in Figure 2. Most represented domain is IPR027417 (P-loop containing nucleoside triphosphate hydrolase) with 1505 annotated contigs, followed by IPR011009 (Protein kinase-like domain), IPR000719 (Protein kinase domain) and IPR002885 (Pentatricopeptide repeat). Among these protein domains/families, “Protein kinase” and its subcategories (such as Serine/threonine-protein kinase and Tyrosine-protein kinase), which are known to regulate the majority of cellular pathways, were highly represented indicating active signal transduction. Noteworthy, top ranked families are also WD40-repeat domain and Cytochrome P450s which play key roles in signal transduction mechanisms, in particular regulating both trichome initiation and phenylpropanoid metabolism [41]–[43]. These findings conform to *Q. pubescens* being well equipped to cope with severe drought stress typical of Mediterranean climate. Pubescence has long been reported to increase reflectance in hairy leaves as compared with glabrous ones [44], and polyphenols, particularly tannins, linearly correlate with sclerophylly in Fagaceae [45]. These morphological and biochemical features, aimed at reducing water loss, decreasing leaf temperature and screening out solar irradiance are of crucial significance in the adaptive mechanisms of plants to the combined effect of water shortage and high solar irradiance [46].

Pfam analysis allowed to annotate 20,448 contigs (Table 2) which contained 3,337 kinds of Pfam domains, among which Protein kinases, WD domain and Cytochrome P450 were highly represented (2,198, 467 and 252 times, respectively). The top 10 most frequently detected domains were all in the above mentioned 30-most abundant InterPro domain list (Table S1).

The eukaryotic clusters (KOGs) present in the Cluster of Orthologous Groups (COG) database are made up of protein sequences from 7 eukaryotic genomes: three animals (the nematode *Caenorhabditis elegans*, the fruit fly *Drosophila melanogaster* and *Homo sapiens*), one plant, *Arabidopsis thaliana*, two fungi (*Saccharomyces cerevisiae* and *Schizosaccharomyces pombe*), and the intracellular microsporidian parasite *Encephalitozoon cuniculi*. Our assembled transcripts were searched against KOG database for in-depth analysis of phylogenetically widespread domain families, in order to predict and classify their possible functions. Overall, 19.94% of contigs (19,146) were assigned into 25 KOG categories, with 3,505 KOG functional terms (Table 2 and Table 3). The three largest categories include 1) “Signal transduction mechanisms” (18.33%); 2) “General functions” (10.73%); and 3) “Post translational modification, protein turnover, chaperones” (9.51%) (Table 3). In the metabolism category, “Carbohydrate transport and metabolism” (5.17%) and “Secondary metabolites biosynthesis, transport and catabolism” (3.92%) were also highly represented. “Function unknown” represented 5.50%, which is quite expected since *Q. pubescens* is phylogenetically distant species compared to ones present in the eukaryotic KOG database.

**Table 3:** KOG functional classification of all *Q. pubescens* transcripts.

KOG Classification	Sequences (n)	Percentage
Signal transduction mechanisms	3356	17.0
Multiple classes	2259	11.4
General function prediction only	1928	9.8
Posttranslational modification, protein turnover, chaperones	1776	9.0
Function unknown	1228	6.2
Translation, ribosomal structure and biogenesis	1065	5.4
Transcription	821	4.2
Carbohydrate transport and metabolism	753	3.8
Cytoskeleton	750	3.8
Intracellular trafficking, secretion, and vescicular transport	747	3.8
RNA processing and modification	723	3.7
Amino acid transport and metabolism	635	3.2
Secondary metabolites biosynthesis, transport and catabolism	604	3.1
Energy production and conversion	603	3.1
Lipid transport and metabolism	540	2.7
Inorganic ion transport and metabolism	445	2.3
Replication, recombination and repair	402	2.0
Coenzyme transport and metabolism	196	1.0
Cell cycle control, cell division, chromosome partitioning	192	1.0
Cell wall/membrane/envelope biogenesis	179	0.9
Defense mechanisms	175	0.9
Nucleotide transport and metabolism	167	0.8
Chromatin structure and dynamics	147	0.7
Extracellular structures	38	0.2
Nuclear structure	10	0.1

### GO classification

**Figure 3:**
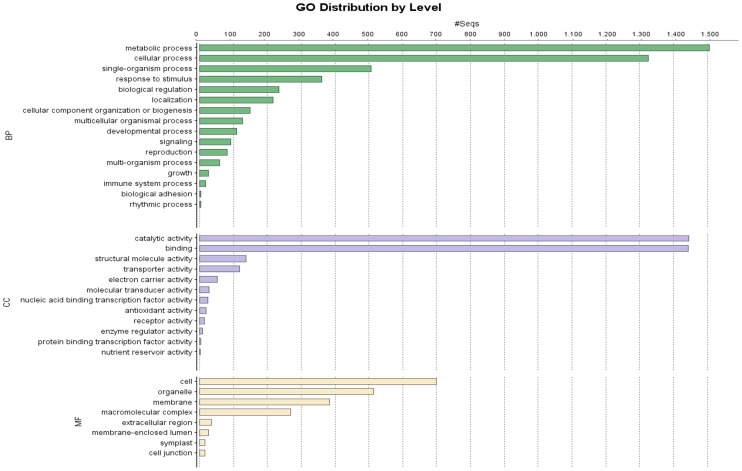
Histogram of GO classifications of assembled *Quercus pubescens* transcripts. Results are summarized for three main GO categories: biological process, cellular component and molecular function.

**Figure 4:**
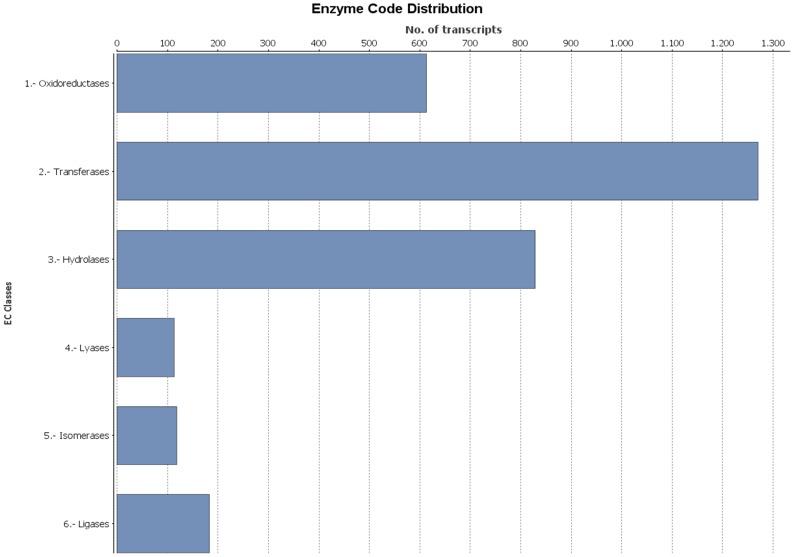
Catalytic activity distribution in annotated *Q.pubescens* transcripts.

Gene Ontology (GO) terms and enzyme commission numbers (EC) for *Q. pubescens* transcripts were retrieved using Blast2GO [30]. Gene Ontology is an international classification system that provides a standardized vocabulary useful in describing functions of uncharacterized genes. Our results show that downy oak differs substantially from model plants. Indeed, just 19,178 (28.1%) retrieved the associated GO terms, and only 8,563 (12.5%) were annotated to a total of 32,844 GO term annotations, of the 68,285 most significant BLASTX hits against the NR plant species database (Table 2). All the extracted GO terms were summarized into the three main GO categories: 15,701 terms (47.8%) belong to the Biological Process class, 5,393 terms (16.4%) fit with the Molecular Function class and 11,750 terms (35.8%) belong to the Cellular Component class. Major sub-categories reported in Figure 3 come from GO level 2 classification. Two sub-categories “cell” (GO: 0005623) and “organelle” (GO: 0043226) occur in molecular function cluster; sub-categories “binding” (GO: 0005488) and “catalytic activity” (GO: 0003824) are clustered in cellular component; and five sub-categories “metabolic process” (GO: 0008152), “cellular process” (GO: 0009987), “single-organism process” (GO:0044699), “response to stimulus” (GO: 0050896) and “biological regulation” (GO: 0065007) were in the cluster of biological process. However, these results assigned only a small percentage of downy oak transcripts to GO terms, possibly due to large number of uninformative gene descriptions of protein hits. Of the 8,536 sequences annotated with GO terms, 2,805 were assigned to 114 EC numbers. In detail, transferase activity (40.6%), hydrolase activity (265%) and oxidoreductase activity (19.6%) were the most represented enzymes (Figure 4). The large number of annotated enzymes within these three groups suggest the presence of genes associated to pathways of secondary metabolite biosynthesis [47], [48], as we detail below for KEGG pathway mapping.

### KEGG pathway mapping

**Figure 5:**
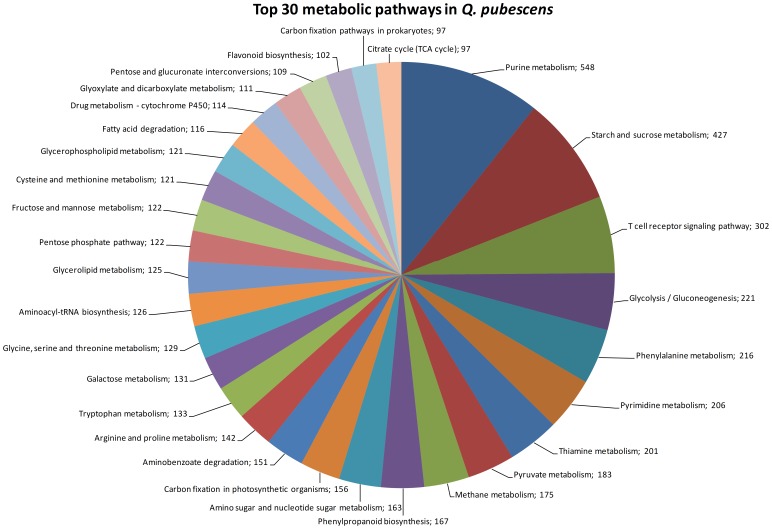
Top 30 metabolic pathways in *Q. pubescens*. This table shows the KEGG metabolic pathways of plants that were well represented by unique sequences of *Quercus pubescens*. The number of sequences and enzymes involved are described

In order to identify the biological pathways active in *Q. pubescens*, the assembled contigs were annotated with corresponding EC numbers against the Kyoto Encyclopedia of Genes and Genomes (KEGG) Pathways database [31]. By mapping EC numbers to the reference canonical pathways, a total of 4,050 contigs (4,22%) were assigned to 138 KEGG biochemical pathways (Table 2). Figure 5 shows the 30 KEGG metabolic pathways mostly represented by unique sequences of *Q. pubescens*. These include “Purine metabolism” (548 members), a metabolic pathway of central significance in plant growth and development [49]. For instance, purine is involved in building blocks for nucleic acid synthesis and a well-known precursor for the synthesis of primary products and secondary products [50], [51]. This is consistent with enzymes specifically involved in phenylpropanoid metabolism (total 485 seqs, 41 enzymes) detected in our analysis. These include both phenylalanine metabolism, the entry point in phenylpropanoid biosynthetic pathway, as well as downstream enzymes involved in general and branch pathways of phenylpropanoid biosynthesis, such as flavonoid biosynthesis. Additionally, others highly represented pathways are “Starch and sucrose metabolism” (427 members) “T cell receptor signalling pathway” (302 members) and “Glycolysis/Gluconeogenesis” (221 members).

Overall, 83,065 unique sequence-based or domain-based annotations using the seven selected database were assigned to *Q. pubescens* transcripts. These annotations provide a useful resource for investigating specific functions and pathways in downy oak.

### Functional Genes Related to Drought Avoidance

To screen functional genes specifically related to drought stress, candidate genes from *Arabidopsis* [32], [42] were local BLASTed against 96,006 transcripts in *Q. pubescens*. A total of 318 contigs homologous to 35 *Arabidopsis* candidate genes potentially involved in drought adaptation were identified in our transcriptome dataset. Overall, 21 putative transcripts regulating the biogenesis and development of trichomes and root hairs have been identified as involved in drought avoidance (E-value, 1e^−5^), and succesfully validated by RT-PCR (Table 4). All amplified transcripts gave amplicons of expected sizes that were cloned and sequenced (Table S2). Programming of epidermal cell differentiation to form trichomes is a low-energy strategy to effectively cope with drought stress in species inhabiting dry and warm areas world-wide [52]. Secretory and non-secretory trichomes serve multiple functions in plants challenged against multiple stresses, as usually observed in Mediterranean areas, particularly during the summer season. As already mentioned, surface appendices are crucial to limit water loss [42], [46] while screening out highly energetic solar wavelengths [53]. Molecular mechanisms of epidermal cell differentiation organs have been extensively studied in *Arabidopsis* [54]. A network of transcription factors, including TRANSPARENT TESTA GLABRA1 (TTG1), GLABRA3 (GL3), ENHANCER OF GLABRA3 (EGL3), which is a paralog of GL3, and GLABRA2 (GL2). GLABRA1 (GL1), which encodes an R2R3 MYB transcription factor, promotes trichome initiation, TTG1 encodes a WD40 protein, GL3/EGL3 encode basic helix-loop-helix (bHLH)-type transcription factors, and GL2 encodes a homeodomain/leucine zipper transcription factor [42], [55]. MYB transcription factors have long reported to regulate differentiation in *Arabidopsis*, such as epidermal cell fate and seed coat development [56]. However, MYBs have been primarily (i.e. in early land plants) involved in the regulation of flavonoid biosynthetic pathway, and following gene duplication and functional diversification (i.e., neo-functionalization) they acquired the new function of specifying multiple cell fates, such as the generation of trichomes formation [56], 57. As a consequence, our study identified a set of genes potentially involved in the biogenesis and development of leaf hairs and phenylpropanoid biosynthesis, thus facilitating disentangle mechanisms of drought resistance in pubescent oak at molecular level.

**Table 4:** *Q. pubescens* contigs related to genes involved in drought avoidance in *Arabidopsis Thaliana.*

Gene name	Sequence similarity	Contig ID	Forward and Reverse Primers 5′-3′	Amplicon length (bp)
Auxin response factor 1 (ARF1)	Arabidopsis thaliana (AT1G59750)	Quercus_contig_413	F: TGGATAGAAGTCTCGCCCAC	706
			R: GCACATTTTCCGAGGGCAA	
Mitogen-activated protein kinase 6 (MPK6)	Arabidopsis thaliana (AT2G43790)	Quercus_contig_575	F: ACAATAGGTTCACTGGGATGGA	907
			R: TACAGTTGGTCCAAGGCCAA	
Rho GDP-dissociation inhibitor 1 (SCN1)	Arabidopsis thaliana (AT3G07880)	Quercus_contig_781	F: TGATGTCTTTGGCTGTTGGAG	749
			R: CTCTGCTCAAGTTGAAGCCC	
Glabra 2 (GL2)	Arabidopsis thaliana (AT1G79840)	Quercus_contig_1106	F: GATCAACCATCCTCACACTGC	2362
			R: TCTCTCTCTTTCTACGCACCT	
Phospholipase D deltaPLD delta	Arabidopsis thaliana (AT4G35790)	Quercus_contig_1195	F: TCTTGCACAGAGGCAGATGA	949
			R: ACACGAATGGAGTAATGGCA	
Can Of Wms1 (COW1)	Arabidopsis thaliana (AT4G34580)	Quercus_contig_1527	F: GGCAAAACATCTCACCAGCA	1888
			R: GGCCTTGCTTTGAAGGATCC	
GL2-Expression Modulator (GEM)	Arabidopsis thaliana (AT2G22475)	Quercus_contig_4979	F: TTCGTGGTTGTCAACAGAGA	474
			R: ACGGTGGATTCGGTGAAAGA	
Phospholipase D alpha 2 (PLD alpha2)	Arabidopsis thaliana (AT1G52570)	Quercus_contig_7695	F: TGTAGTCAGATTTGGCACCCA	2360
			R: CGTCTATGAGGTCGACAAGC	
Cyclin D 3;2 (CYCD3;2)	Arabidopsis thaliana (AT5G67260)	Quercus_contig_11597	F: GGGGACTTGGACTTGAGTGT	880
			R: GCACTTCCAAAACCACACCA	
Transparent Testa Glabra 1 (TTG1)	Arabidopsis thaliana (AT5G24520)	Quercus_contig_11685	F: GGACTTCGAGCATTGACACC	417
			R: GAGAATCATCCCCAGCCGTA	
Phospholipase D beta2 (PLD beta2)	Arabidopsis thaliana (AT4G00240)	Quercus_contig_15358	F: GCCACACGAGCTCATTCAAT	2523
			R: CCTGAATGGCAAGAAATGAACC	
Cyclin D 3;3 (CYCD3;3)	Arabidopsis thaliana (AT3G50070)	Quercus_contig_21908	F: GCACTTCAACAGACAGACGA	1085
			R: TCTGCAAGAAGAGGAAACCCA	
Cyclin D 3;1 (CYCD3;1)	Arabidopsis thaliana (AT4G34160)	Quercus_contig_22955	F: AGGTTCTGTTTAGCTTCCTCCT	1038
			R: TTGACTACGCGGTTTCAAGC	
Phospholipase D alpha1 (PLD alpha 1)	Arabidopsis thaliana (AT3G15730)	Quercus_contig_25616	F: GTCAGGCAAAGCAGAACCTC	1157
			R: TTGCAAGTGGTGGCTACAAG	
Enhancer of Glabra 3 (EGL3)	Arabidopsis thaliana (AT1G63650)	Quercus_contig_32077	F: CTGTGAGAAGCATTCAGTGGA	207
			R: CTTCACCAGCTGAGAGGGAC	
Werewolf (WER)	Arabidopsis thaliana (AT5G14750)	Quercus_contig_35143	F: GAAAAGGGCCTTGGACAGTG	332
			R: GGTTTTCCCTTTCTTGATCCCA	
Root Hair Defective 2 (RHD2)	Arabidopsis thaliana (AT5G51060)	Quercus_contig_39550	F: GTTGCGGTTCACAGTGTTCA	2451
			R: TCCGACTCGTGTTTCTGGAT	
Triptychon (TRY)	Arabidopsis thaliana (AT5G53200)	Quercus_contig_41116	F: GGAAACAAGCCAAGACCAGG	211
			R: GCAAATACCTCACCATGTCTCA	
Phospholipase D epsilon (PLD epsilon)	Arabidopsis thaliana (AT1G55180)	Quercus_contig_47871	F: CCCTAACACCTTCACTTGCC	186
			R: ATGAGCCCCACAGATGTTGG	
Glabra 1 (GL1)	Arabidopsis thaliana (AT3G27920)	Quercus_contig_56291	F: GATGGAGGAAGGGAACACGT	351
			R: TTTTGCTTCTTGAGGCCCAG	
Cyclin A2;3 (CYCA2;3)	Arabidopsis thaliana (AT1G15570)	Quercus_contig_72031	F: CAGGAGATTCATTCAAGCAGCA	248
			R: CAAGGACGGTGGTTTTCAGT	

### Simple sequence repeats (SSRs) and SNP detection

**Figure 6:**
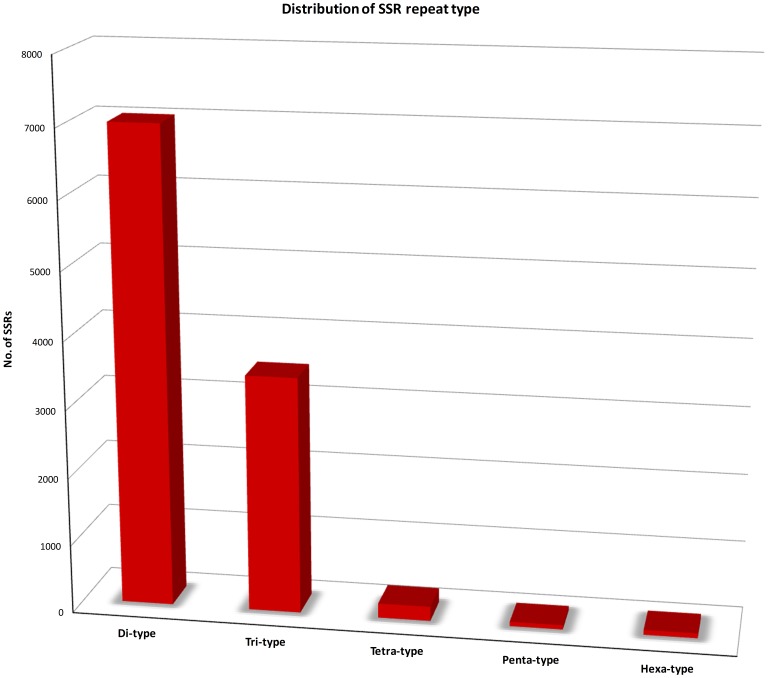
SSRs distribution in the leaf transcriptome of *Q.pubescens*.

Simple Sequence Repeat (SSR) markers, also known as microsatellites, are short repeat DNA sequences of 2–6 base pairs, which are important for research involving population genetic structuring, demography, relatedness, and the genetic basis of adaptive traits [58], [59]. In this study, a total of 14,202 putative SSRs were identified in 96,006 assembled transcripts, of which, 3,366 were compound SSRs. The SSRs included 7,011 (49.3%) dinucleotide motifs, 3,478 (24.5%) trinucleotide motifs, 212 (1.5%) tetranucleotide motifs, 65 (0.5%) pentanucleotide motifs and 72 (0.5%) hexanucleotide motifs (Figure 6). The most abundant repeat type was (AG/CT) for dinucleotide SSR and (GAA/TTC) for trinucleotide SSR. The observed frequency of SSR (14.8%) was slightly lower than that to that observed in related *Quercus* spp., (18.6% and 23.7% in [60] and [28], respectively). Surprisingly, di-nucleotide repeats were the most common SSRs in our transcriptome (49.3%), with tri- and tetra-nucleotide repeats being present at much smaller frequencies, in contrast to the most frequent motif (tri-SSRs) found in *Q. robur* and *Q. petraea* [28], [60] and in agreement with *P*. *contorta* [24]. Based on the 14,202 SSRs, 10,864 primer pairs were successfully designed using Primer3: information on the contig identification (ID), marker ID, repeat motive, repeat length, primer sequences, positions of forward and reverse primers, and expected fragment length are included in Table S3. Twenty microsatellites were randomly selected (15 dinucleotide and 5 trinucleotide SSRs) for PCR amplification in two individuals: 17 (85%) were effectively amplified producing fragments of the expected size, validating the quality of the assembly and the utility of the SSRs herein identified (validated primer pairs are highlighted in Table S3). To confirm marker usability and characterize the selected seventeen SSR markers for variation a total of 8 individuals from two Italian populations (four from Spello and four from Volterra) were analysed. All selected SSRs displayed consistent patterns, eleven loci were polymorphic and six monomorphic (the absence of polymorphism might be due to the small sample size). Primer sequences, repeat motifs and detected alleles are shown in Table S4. Similar research carried out using Illumina sequencing technology in sesame showed that about 90% primer pairs successfully amplified DNA fragments [39]. High-throughput transcriptome sequencing showed to be superior resources for the development of such markers not only because of the enormous amount of sequence data in which markers can be identified, but also because discovered markers are gene-based. Such markers are advantageous because they facilitate the detection of functional variation and the signature of selection in genomic scans or association genetic studies [61], [62]. Transcript-based SSRs are advantageous compared to SSRs in non-transcribed regions owing to their higher amplification rates and cross-species transferability [63]. Currently, although many SSR markers were identified in the Fagaceae family, only a few SSR markers were reported in *Q. pubescens* [64]. The predicted SSRs from the assembled transcriptome of *Q. pubescens*, will likely be of value for genetic analyses of *Q. pubescens* and other related non-model plants.

Single Nucleotide Polymorphisms (SNPs) were identified through the analysis of the multiple alignments produced during the assembly process by using CLC Genomics Workbench. The analysis of 96,006 contigs resulted in the in silico discovery of 19469 variants, including 18,425 (94.6%) SNPs, 858 In-Del, Insertion-Deletions (4.4%) and MNV, Multiple nucleotide variants, 182 (0.9%) (Table S5). The variants were distributed in 8652 contigs (9.0%) corresponding to 15,752,655 bp, 26.6% of the total *Q. pubescens* transcriptome. Ten SNPs, 8 from different contigs and 2 from the same contig (Figure S2) were confirmed by cloning and sequencing fragments ranging from 151 bp to 557 bp amplified in the four genotypes used for the transcriptome assembly. Newly designed primer pairs and fragment sizes are listed in Figure S2. The polymorphisms were confirmed by detecting at least one variant for each SNP in the four genotypes (one chromatogram for each genotype is reported in Figure S2, labelled as sample 1 to sample 4). The frequency of the variants resulted equal to 1 variant per 6,854 nucleotides on average, lower than that observed in other forest tree species [24], [48] that could be explained with the limited number of genotypes analyzed and the strict parentage relatedness that reduced the possibility of SNPs mining.

## Conclusions

In the recent years, transcriptome sequencing became a most powerful and efficient approach to uncover genomic information in non-model organism [20]. The *de novo* assembly and annotation of the *Q. pubescens* transcriptome provided complete information concerning the expressed sequences of leaf tissue. Data of our study represent an important tool for discovering genes of interest and genetic markers, thus allowing investigation of the functional diversity in natural populations. Our study (i) generated 318 milion raw nucleotide paired-end reads, comprising 96,006 transcripts from *Q. pubescens*, (ii) identified putative function in 83,065 transcripts for the species and several genes related to numerous metabolic and biochemical pathways, (iii) discovered a large set of high quality marker motifs and variations (14,202 genomic SSRs with designed primers, and 18,425 higher confidence nuclear SNPs) which could be used for generation of polymorphism based analysis within species. Detection of functional variations and the signature of selection in genome scans or association genetic studies are facilitated by these markers.

These tools will be crucial for future comparative genomics studies in other *Quercus* species, taking advantage of their remarkable ecophysiological differences. Our characterization of the leaf transcriptome in *Q. pubescens* has not only enriched the publicly available database of sequences for members of the *Quercus*, but will also facilitate genetic analysis of other non-model organisms. Furthermore, our data demostrate that Illumina paired-end sequencing can successfully be applied as a rapid and cost-effective method to non-model organisms, especially those with large genomes and without prior genome annotation.

## Supporting Information

Figure S1 **Flow diagram of whole transcriptome analysis for** ***Q. pubescens*** **.** The steps and sets of sequences involved in Illumina sequencing, assembly of reads into contigs, annotation using protein databases, and genetic marker discovery and characterization.
(PNG)

Figure S2 **Validation of ten predicted SNPs.**
(DOCX)

Table S1 **BLAST hits from the NCBI Viridiplantae Nr database, NCBI NP RefSeQ database, UniProt database and KOG, Pfam and InterPro domain/families assigned to** ***Q. pubescens*** **contigs.**
(XLS)

Table S2 **Sequences of** ***Q. pubescens*** **genes related to drought avoidance.**
(DOCX)

Table S3 **List of** ***in silico*** **SSR primer pairs derived from** ***Q. pubescens*** **transcriptome.** Validated primer pairs are highlighted.
(XLS)

Table S4 **Characteristics of the** ***Q. pubescens*** **microsatellites markers analyzed.**
(DOCX)

Table S5 **List of single nucleotide variants derived from** ***Q. pubescens*** **transcriptome with sequence annotation (BLAST hit, GO and EC).**
(XLS)
