# Supplementary figures and images for: RNA-Seq Analysis of Quercus pubescens Leaves: De Novo Transcriptome Assembly, Annotation and Functional Markers Development
Source: PLoS One. 2014 Nov 13;9(11):e112487. doi: 10.1371/journal.pone.0112487 (PMC4231058; doi:10.1371/journal.pone.0112487)

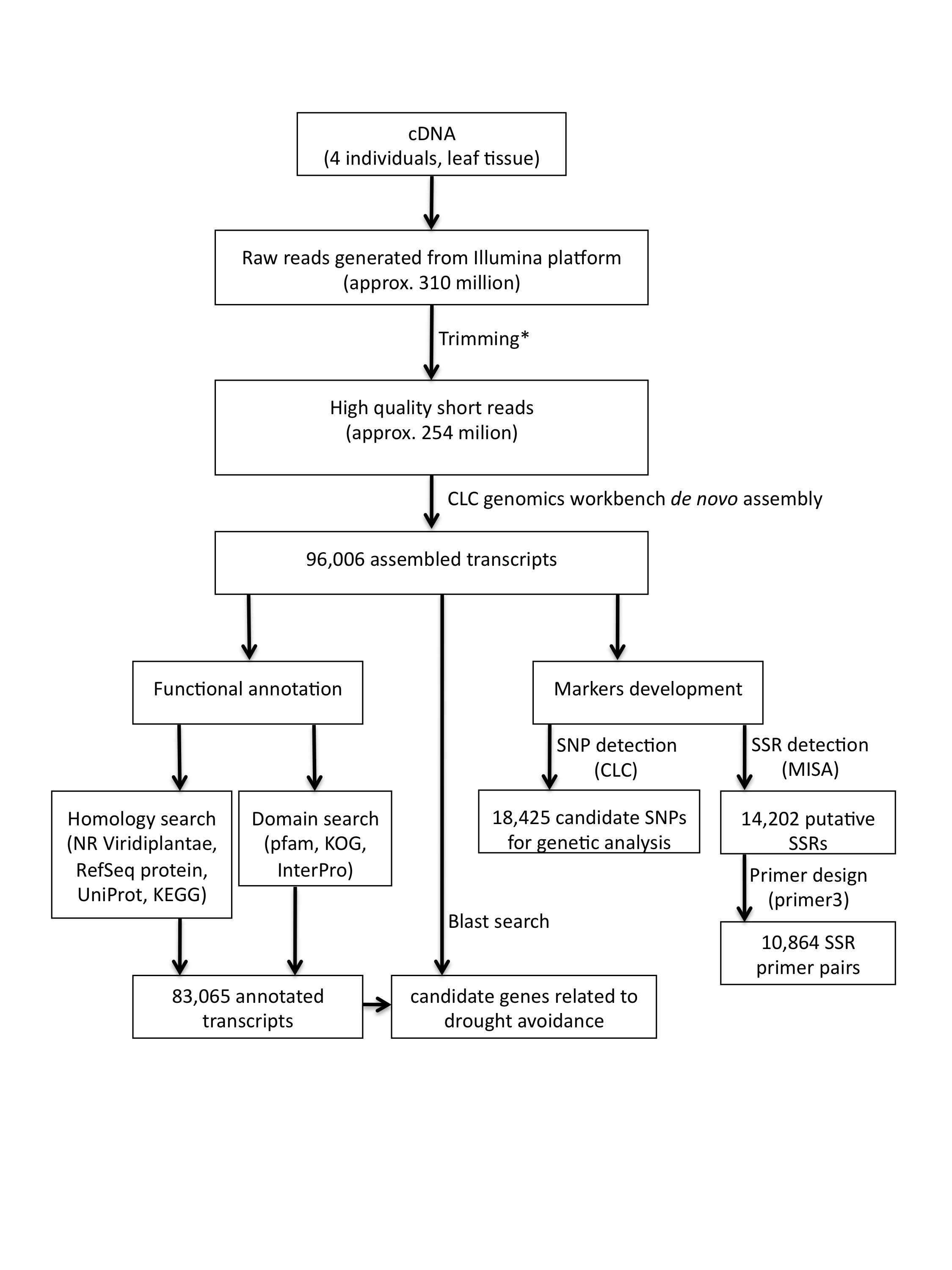

Supplement: Figure S1 — Flow diagram of whole transcriptome analysis for Q. pubescens . The steps and sets of sequences involved in Illumina sequencing, assembly of reads into contigs, annotation using protein databases, and genetic marker discovery and characterization. (PNG) [file pone.0112487.s001.png]

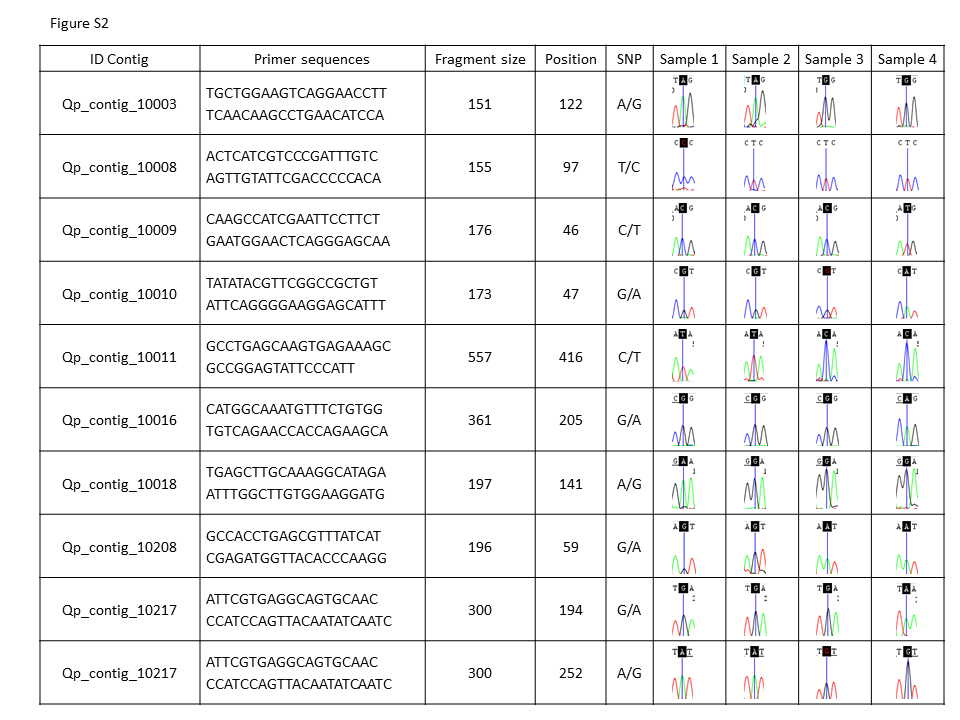

Supplement: Figure S2 — Validation of ten predicted SNPs. (DOCX) [file pone.0112487.s002.docx]
