# Supplementary material for: RNA-Seq Analysis of Quercus pubescens Leaves: De Novo Transcriptome Assembly, Annotation and Functional Markers Development
Source: PLoS One. 2014 Nov 13;9(11):e112487. doi: 10.1371/journal.pone.0112487 (PMC4231058; doi:10.1371/journal.pone.0112487)
Supplement: Table S2 — Sequences of Q. pubescens genes related to drought avoidance. (DOCX) [file pone.0112487.s004.docx]

**Table S2.** Sequences of *Q. pubescens* genes related to drought avoidance (Table 4). Each of 21 predicted transcripts was amplified in leaf tissue with the underlined primers (listed in Table 4).

>Quercus_contig_413

TGGATAGAAGTCTCGCCCACGTTGTGGCTCACAATATGAGAAAGCAGAGGGCTCAACTGGGGATTTCCACGCACCAAGTACAGGAAAATCTGGTGTTTGTGAAGGTAAAACAGCAGGCCGTGCCCGCTTGTTCCGTGGTGTGGGTTGCGAGCTTGGTGGAGAGTTTGCCACAAGTGGCTCCAATTCCCATGGTGAAACTCTATCTGGACGCACAATTGATGAATGTTCATCCCACTGGACCTTTAGTGATCGCCACTCAGAATCAGTCCATCCAGATGATTTATTGTCTTCAACACCAACAATTGTGCCACTGAACCTTCTTTCAGGAACTTCCTCACCCTCAAATCTCATTCTAAACCTCATCCCAACAGATAGCTTATGGCTCCGAGCTTCAAGGTACTTGTTCACACTTACAATAAACTCCGACCGACTTGTTCTTGGCTTGTAGAATACAGAGAAGAGTGTTTTAGTTGCAATGGCATGAGATGCAGTGGCTAGAACCCCAAGATGCATGCTGTGACTAGATATAACAGAAGATGGCATGTTGCTTAGTTGTCTCATGAGTCTCCTTACTCCAACGCGCAGCTCCCCATTTTCACCTCTCAGAAATATAAATGCATCACCAGCCACTAATTTTTTGGAACTAACAAAGACACTCCATCCAGTTGTCAGCAAGTGGCGCCTTGGTTGCCCTCGGAAAATGTGC

>Quercus_contig_575

ACAATAGGTTCACTGGGATGGAAGACACACTCATTGACTGAACCAGTGTGGCCAGGGAGCTTATACAAGATGCGTCTAGATGTAGTATCCCAAATGTAAACCATCCGATCTGAACTCCCGGCAGTGACTTTGCTTCCATCAGGTGACCAGCTACACTTCAACAAGTTCTTTTCAAAGTTGTGTTGGTGCCCTTCCATTACCTTTACACACCGATTTTGTGGTGCATATGGACGCATATCCCATATGCAGAGTTTGCAATCCATACCATTTGTAAGAAGATAGGAGCCATCAGGACTCAACTGCATACCTGTTATCATATCTTGATGACCTTGAAGTGTCATTGTAACTTCACCTTTGCGCAAGTCCCATACCTTTACTTCATTGTCAATACCACCTGTGAAGATCTTATCAGATGCATCTGAGAAACCGACGGCTGTGATTTGGTATTTATCTGGAAATGTCTGAATGGCACCCCTCTGACGCATATCCCACAGTTTAGCAGTTCCGTCATCAGAGCCACTCACCACAAGAGGTGGTCCCCTTCGGGAAGGACAACATGAATTAACATGAGGAGTGTTCTGCCATTTTCTTGATTTGTTTTCCTGTTTCAACATCCCATGCCCTCAGCGTTTTGTCAGGGCTGGCTGATATTATCTGTGATCCATCAGTGGTCCAATGAAGATCCAAAACTGCATTCTTGTGCCCTTTTAAGACCATGAAGTTCTTGCAATCCCCGTGCACATTCCACAGAAAAATTTCTTTGTCATGAGACCCAGATGCAACAACTGTTCCCCCAGGATTGAACTTCATTGTGTATATGGCACTCTGATGGCCTGTCAGCAACATGATTGGCGATTCCAAACTTGATGTCCGCTGTTTTCCATTTGGCCCAGGTGCTTGAGGAGCACTATATGGGACAGTAGACCATTCCATTGGCCTTGGACCAACTGTA

>Quercus_contig_781

TGATGTCTTTGGCTGTTGGAGCTGTTTCAAGTTCCAAGAACATGGGGTTTGATGAAAACAAGGAGGAAGGAGAGAAAAGTACAAGAGAAATAGAGGAAGCAAAGCAAGAGGGTGATGATGAGGGAGGGAAGATTAGCAGACAGATGAGTGAAGCTTCTCTATATGCAACTGATCTTGAGGAGGAAGATGACGAGAGAAGCGAATTACAGTTGGGTCCTCAGTACACTCTCAAAGAACAGCTTGAGAAGGATAAGGATGATGAGAGTCTGAGGAGGTGGAAGGAACAGCTTCTTGGGAGTGTTGATCTTAACAATGTCGGAGAAACTCTTGAACCAGATGTGAAGATATTTTGTCTGTCAATCATCTCACCTGGTAGATCTGACATTGTTCTTCCTATTCCTGAAGATGGAAAGCCCAATGGTACATGGTTTACATTGAAAGAAGGTAGTAAATACAAATTGAAATTCTCTTTCCAAGTGAGTAATAACATTGTTTCTGGCCTTAAGTATACCAACACCGTCTGGAAAACTGGTATAAAGGTGGACAGTTCAAAAGAGATGATTGGTACATTCAGCCCTCAGATAGAGCCTTACGCACATATAATGCAGGAAGAGACCACCCCTTCTGGCATGTTTTCCAGAGGATCATATTCGGCTAGATCAAAGTTTTTAGATGATGATAACAAGTGCTACTTGGAGATCAACTATGGCTTTGATATCCGAAAGGAATGGGCTTCAACTTGAGCAGAG

>Quercus_contig_1106

GATCAACCATCCTCACACTGCAAGCTTGTCTTAATGTTTCTTAGGGTACATGATATTAAAGTGTTAACAGACTCCACAGACTCCACAGAAAGCTTTGCTGTGGGAGAGGTATTAGTTAGGATCTGGATTGCTATTGTAAGCAAAGACCCTCCTTCTGTGCTCTTCTCCTCTTGCCTAGATGTAATCACTAAAGGCCTTGACTCGACCCCATCTGGAAGAATCGAAAATCCTGAAGGTAAGATGGCCATGTTGCTAGAGTCGCATCCTGTCATCACAGACTGCATGCCGGTAATGTCCACTGGAGCATAGACCACCATGGACTCGTATGCATTGGTGCAGCCATCTTGAAGTACCCACATGCTGTTCTCTTTTGATTTCATCGTCTGAATGGCCACTGAATTGCCACGATCTTGCCCTTTAGCTAAGTTTGCAATGGATTGTGCTGGACCTCCACTTGACATTATATCCCACTCACTCCGGTGAGCTTCGTCTCTTAAGAAATCGAATAGGACATGAGGAGAGACAGGCAACCATACAGAAGAAACTGCACACAAGATCACCCCAAGAGGTTCTCCAGGGTCATTCAAGTTCTTCCTAGAAGTTATCCTGATGTCTTCCCCTGTCTTACTTGACACCTTGGTCCATGTATGATAGCTTGATGCTCCAATTGCTCTGCAAAAATTCCATGTCATCCTTTGTGCCAACTTCAAAATGCTTTTCCTCCCGGCCAGTGTGGCAACACCAGTTGAATCCTTCATGGGAACATTGGTTGCCATGAAGAAAACGAGCCGTTCACATTGGAGTTGCAGTGTAGCCATCCAATGCCTTGCACCAAAGGCTAGACCACTGTTAACAATAGTGCGGTACATGGTATGAACTGGACTTTTCTGGCATTCCAAGTGCTCTACCCAAATCACCTTGCAGTGGCCATTTGATTTATCCTCTATGATGCAACCAGATGGGCGTTTTCTGCATTTCACAAGGGATGCATCAATGTTATCCTCAACTTTTTCTATAGAAACATCCACAATGGCCCATTGTTCAGCACTCAATTGCTTGCAAAATCTGACGAAGTACACTTCTCTAGTGGGTACCATTGGTGTGAGCATTTGCAGTTCTGCAAACATAAGTTGTACAGCGCCATTTTTATTAGGACCTTCTCCATTGGAGATAACATCAACGGTGGCTGCCTTTGAGATCAAGCATGGAAACATTTCCTTCCATTGATTCACATCCATGAAACTTTGAACAAGACGAGGGAGATCCACAAAAACAACTCCAGTCTCTCTTGATGCCTCAATGGATCTCTTTGGCCGCGCATTGCTTGAGTTTTCAATAGAGAATTCTTTCATGTACTCATCATAATTAAGTATTTCCCGACCAGTCTCAACACTTCGAATCCATAATGGTTCTCCTGCAGTAGCCATCTTTGTGAGCTCCTCCATTGCTTGATTTACTATCTCCATTATCCTCGTCTTCTCAATCCCGAAAATCCCAGTGTAAAAATCCAAAGAGCTTCTATTCTCTTGGTCATTGCCAGCTGAACATGAAGGTGAGGATGCCCCTGGAGGGTATTTCCCTAGAGCTGATCGGAGTTTTTCTACCTCAGCTTTGAGTCTGGCATTTTCAATTCTGAGCTGTTGTTCCTCCGTAGTCAAAGCAGCTTCTCGGCTAGTGGTGGCAGTGCCACAATTGGGACAGCAAGCTTTATTTATTTGCTCCCTCATGGCTTTGTTTTCGTCTCGGAGTTTCTCCATTTCTGTCTTCAACAAAGAATTCTCATGGCGCTCTTGTATAGCCTTAATTTGGGTTCGACGATTTTGAAACCAGAACTTGACTTGCCTAGGAGCAAGGCCTAATTGCTTGCTTAGTTGTTGCCTTTGCTTCTCATCTGGGTGGGGTGACTCTTTAAATAGCGCTTCCATTTCTCTAATTTGCTCAGCAGTGTGCCTATGATACTTCTTTCTCTTCTTCTTTTTGCTCTTGTCTCCGTCAGCACCATCATCATCAGGCTCTCCTTCGCCGTCAAATTCATCGTCGGACCTTGACCTCAGAGGCTCCGAGTTTTCACTACTAATCTCCACCATGTCATCTCTCCGGCCACCTCCTCCGCTTCCTTCATCCCCTTCTTCCACCTCCATGTTGGGTGTGGCCGCTGCCCCGGCATCGCGAAAAATCCCGGCAAGGCTGAGAGAGAGAGCTGGAGAGGCAAAGAAGTCTTTGGTACGAGAAGAGGGTGGATTATTCGACATGTCCACGCCCATGGCAAGAATGAGAGTTCTTGGGGTGGCAATGGCAAATGGGTTGTTTTAATAAGGACCAAGCATGTTGGTGGTGATGAAGGTGCGTAGAAAGAGAGAGA

>Quercus_contig_1195

TCTTGCACAGAGGCAGATGAGGGGGCACCCTCAGGCCACATAGGTATGACAATGTAAACTGCAAATTTCTCCTTCGCTCGAATCTTACTAGCAATCTTTAATGCCAGCTCCATTGGAATTAAATTATCGGCACCTGCATATTTGTAATTTGGCCATGCATATGAAGATCCAAGAAAATATTGGTTCTCAATATATATAAAATGTTGAGCAGATCTGATTGCCTGGATGTATGCTGTTTGGATACTCTTGTCTATAACCAAATTCTTTTCACAAACAAGATTCTGAGCTTCAGCATCATATACAGTTTTAGGAAATCCTTTCAAAGACCCTGAATCAATTGAGCGGAAAACCTGAACATGCCAGTTTTCAGGATCATCTTCCTTGGAAACACATATTATAGGGTCGTCATTTGGAATTGATTTGGTAGGACTAAGTATCCACGAGATGCGTTCTAACTTTATTAAGGCATCATCATGCCAACGAGATACCCTTTTGAACCGCTGCGACAACTCTGACCATTTTGTTGCCTTTCTCCAACGCTGCTCAAAATTAGTGAGCACATCATATGCAGCAGGCCCTTCAATTTTACAATGTAAATCATGCCATGGTTGCCTTGGACCCTTAGTTCCCGCAGAAAATGTTGGGTTGTGATAATCATTCTCATATACAGTGTCCAGATCACGAAATAGTCGATGCTCAGGAGTATCATAGCGGCCATCGCAAAGGTCTAGACCTCCTATAAAAGAAGTAATCTTCCTGTTATTTCCAGACGCTTGTGTATCCACAATCACACATTTCTGATGATGCGTAAAAAGGGTTCCAACGACCTGTTGTTTGAAAATGCTAAGCTTACTGCTGGCATATCTAGGTGACAGAACACATGTAACCGAAGAGTGCTTGAAAAACTTGCGAGTTTCTTCATCATGAGTTGCCATTACTCCATTCGTGT

>Quercus_contig_1527

GGCAAAACATCTCACCAGCAAAAAAACTTCTTTTTCCGCAACTTTTCTTCCTCTTGTCTGTCTATGTAAGCAAGTAATTCTTCTTGTCTCATTAAAGCATCATAGAGAGCCTGTTTAGTTGCAATTAGCTCTGCTTCTAGGGCATCCACACGACAGACAGCAGCATTAAGCAATTCCTCTTTCTCACAGGGCATCTCAGATGGCTTTGATTGGAGCATGGTGACCTTCTCTTCAAGCTCACCAAGCTTGTTCAGCACAGAAGACAGGAGCTCTGCCTCTGTGTAAGATGGAGTTTGTGAAGTGGGGTGAAATTCCTCTTTGTCTGTTGCATCAGGAGTATTTTGCTCAGAATTAGGTAGTGTTTCAGGAAGCTTATTTGTCACATGACCAGCCACTGAACGGAAGACAGTGAAAAAGGTCATGAAGAAGGTCATAACAGCCACAAAAATATGACCACGAATCCCTTCAGGTGGCTTTTGGTTGTCAGATGGGGGAAGAGTCCCTTTGGAAGCAACGGTTGCACAAGGTTTCTGGAGGGACACTTGCTTCTTCCAACTAGCATCGACAGCCTTGTCAACCATTGGAACATATTCATCATACCCTGGGAAGCTACTCGCATAGCTTGCCTTTCCAACCACCTTAGATTCTTCACGGACAGGAGTCAATCGAAGGTGTGAATAACTCCTATTTGCTTTTGGGGAAGTGATGTCTTCAGCTTCAGATCCTGATTCAGCTGTGGATGTATCACTTCCTTTTAACATTGGGCACTGCGGCTTAGCATAAGCAATAACCTTCCCCTCACTGTTTAAAACTTTCACAACCTGCCTGGCACGCCGTGCTTCACCATTAAGAACCATCTTTAAAATTTCAGGGTTTTTCCATGGCCCCTTGTCAGACCGGAGGCAACCTCCCTGATCTGCACAGGTACAGGTACCACCCAGAAATTCTGGCAACTCACTGGCATCAATTATCTCAAGCAATTTGCTCTGGTATTTGTTTCCAAGAACATGAATCTTGGAAGTTGTTTTCGGATCTAGAAAAGTCTTGACAGTATTCCATAGTAGCCTAAATCCAGGGCCAGCATTGATGATAAACATCTGGCAAAGAGTCTCTGGATAATTGTCACCATCAATCTTCTGCAACCGCATGATGAGATCCCGTGCAGATTTTGTAAAGTTCTTAAAGCCCACGCCTTGAACATCTAAAATTGTAGTGCTTGAATCTATGTGCCTCTTTGCAGCAATGGTACAAGCTGGAAACTTTATGGCAAAGCCTTTCTCAAACTCCCGCACATGATATTTTACATACCGATCCATGGTTGTAACTTGCATAAGTTTGTTAGGATCAACCCTTCCCAGTCTTTCAATATAAACAGGTCTTCCTTCCTTGTCCACACCATGATGACCATGAGGATAATATTTTAAAACTTCATGCAACTCTTTGAACTCGAAATCCTCCATAATAGTGTCAGCACCAAATTCCTCCCTCCACTGAAGCATATCGGCCCACATATGCTTTGCTTTTTCGATATCAAACTTCCTTGCTTTTAAAAACCTCAACATCATATGGTAATCATCATGTTTTTCCGGTAGCAATTCATCCATTATGAGTGATTGTCGAAATCCATCAACGGCCTGTAGCTCCTCAACATCCCTAACATCCTCAATGGAAACAGAGCTGACTCGGCCATCACTTTTTCTTCTACTGCTCTTCTTCTTGAGAGAGTGTTTAAATTTGGTAGATGCATTGAGTGCTTTCTTTTTCAGAGACCCCATTCTGGTCCTCCTTTCATCCTCAGAGTTTTCAAAATCAGATTTCCTTTCTCTTCTCTCGTCTCTTCTCTCATCTCTTCTCTCATCGCTACCAGAGGATCCTTCAAAGCAAGGCCTAGCGAAGCGATCGAGTGGACCCGAC

>Quercus_contig_4979

TTCGTGGTTGTCAACAGAGATAACCTGTATGTATTTTTCCCCTGGCTTGACTTTGCTTGTTGACGGATTCACTGCCCTCAACTGATGTAATGGAATAACCACCTTGTAATAGCTCCATTCAGTCTGGTCACCAGTTTTGTAAGAAAGTGGATTGTCACTACAAAATGCAAGTTTTGCTGTGGACAGATATAGAGTTCCCATTACTGGTCCAGCAGATGTGGATAGATAGGCAGCATATGTCTTTTGAAGCTTCTCCTCAGGCACAGTCTCAAAAGTTGATCGAAAGATCTTCTCATAACCACCTTCTGCAAGAACTTTTGTTCCTTGAGCAATTCTTCCCACAGCAGCATCAGCAAAACTAGGACCCGTTTTCAAGTGTTGCCACATGTTCCCAGCGAGATCCTCAGCCTTCTTGGTTGCCTCCGCCGCCTTCTTCCCCCATTTCCCAAGCACATCTTTCACCGAATCCACCGT

>Quercus_contig_7695

TGTAGTCAGATTTGGCACCCAGAACACGAGCCTTGGTGTCAGGGAAGAACTCAAATCCAGGCAGCTCTGTGACATTTCCTTCACTATCAACCCCGACAGGGTAACGCAGCAAGTGTCCTGGAAGGTCATGTTCAAGTGTCTCACTTGAATACAGATCCCAATATTTGTCAGAAATCTGGTTCAACTTCCTGATACACTCCTCGCTTTTAGGGTGTAAGAATGAATCATCCAGCATGCCAAGGTGCTCATACCATAGTGACATACGGAAACCATGGATCTGGCCACGTGCTGGCTGCCTGTTAGCCAGATGATATGGTTGGTAAGCTCCCATTGCTATCTCAGAGTCCCTAGCTCCATCCATTGATCTCTGATTGATGTTGGCAGATCCAATTATTATATATTCATCATCAACAATCATCATCTTAGCATGAACATAGATCATGAAGCGCCGGGACTCCTGAGCTCGACTATAGTCTGAATCAGGTGGTGGTTTTTCTGATGGTTCATATTCACCCTGCTTTTTCACCTCCCGATTTCCAAGGCAGAAAAATGTCAAATAATTCCGAGGATCCTCATCAATACCCTTGGCTTTCAGAGCCTGAATAATATCTTTATACATCATATCCATTGTCCTCCTCTGCCAATCTAATATGGCCTGAACTGATCCACTCTCCGGCATACCCTCTGGCCACATCGGGACAACAACATAGACAGTGAACCTCTCCCCTGCTTCAATCTTGCTGATAATCTTAAGTGAAAGCTCCTTTGGAATCAGATGCAGAGCACCAATATCCTCAGGCTTGATATCATCAGCACTCCAGCAAAAAGAGCTTCCAAGGAAATACTGATTCTCAATATAAATGAAATCCTTTGCACGTCGAATAGCATTAATGTAAGCATCCTGAATGCTTCGGTCAATGATATTATCCTTCCCACTGACAAGCCCAGCTCTGGCTGCATCTTCAGGTGTCTCTGGGAAGCCAAAAGCAGCCCCACCATCAATGGATCTAAACAACTGCACATTCCATGTCTCATTGTCATCTGGAAACATAACTGGAGATGGGGGGATGATGATTTCGTCAAGCTCTCTAAGCTGAACAAGTAAATCCTTACCACCTTGCTTTCTCCACCTCTGCTCAAAATTAAATAAGACATCCCAAGCAATTGGTCCTTCAAGCCTGGAGTGGATATCATGCCACGGTTCCCTTGGACCACCTTTTGTAATTGAAGCTCCCTCAAAATTTGGCTGATGAAAATCATCATGATGTGCAGTGTCCAATGTCCTGAAGAGTGAATGAAAGGGGGTATCATATCTCCCATCACAAAGATCAATACCCCCAACGAAACTCACAATTCTCCTCTTTTGTGATCCTCCACTAGGCATTTCACTGTCCACCACCACAATCTTCTGGTGATGAGTGAACATGGTTGAAATCGATAAGTCCTGAACAATGCTTCCACCATCATCGGGATTTCGGGGACACAATACACAGTGCACGTCAGTATTCTGGAAGTAGTGTTCAGTTTCTTCATCGTGGGTGGCCATCAATCCATCCTTTTTCAGTAACCCAACAGAAGTCCTGTCATCCCAAACAAGCATAAGAACCCTAACACCTTCACTTGCCTTTTTCTTAAGCAGATCACCAAGTAAGATGTCTCCTCCAGACTTTGGCCTCCTTGAGTCCCTCACCAAGGAAATTTCAGTATAGACCGACCATCCAGTAATGTAGATCAAGTGTTTTGCATTAGTGATTGCATCAAAAATGTCTTCCCAACATCTGTGGGGCTCATAATATGTGCCTCCAGCAAGAGGGATTTTAGGAACAAATTTGTCTGGGACATGAGCATCTTGGTACAGAGAAATCCTACATCCTTGCCTCTGTGAGAAGAACGTGTATGGCACTCCAGGAAATTTAAGACTTCTAATACCCCGAGCCCAATTATGGTCTTTTGCAACATCAAAATATTGTAGCTTCACATGGATCTTCGAACCTACAGGATTTTTCTCTTTATCCAATATATCAACCCATCTATCCACTTCCTCCCCATCTAAGAGTTCTTCAATAGGTACATATGCTCTTCCAATTAAGGTTGCCCCAATAGGATTATCGTCTTTGACAGTGAAAATAACATTTGAAGCCAAATGAGCACAGTAGATATGAAAAGACTCATACCACCTGGGATTGTTAGGCTCATTTTCTAGTATCCTGGTCTTCCCAACTCTCGCCTTTTCTAGATCAATGGTTGCGTAGACTTTAGGAACTCCTTTACCAATACCAACTGTCTCTTCAATGTTTGCCAGAATCTTGCGGAAGATCTTAGGGCCACCACCACTGTGGAGCTTGTCGACCTCATAGACG

>Quercus_contig_11597

GGGGACTTGGACTTGAGTGTTAGGAGACAGGCTCTTGATTGGATTTGGAAGGCTTATGATCATTACAGTTTTGGACCATCAAGCGTGTGTCTATCCATGAACTACTTGGATCGCTTCCTATCAATTAATGAATTGCCAAGAGATAAAAGTTGGGCTGTGCAATTGTTAGCTGTAGCTTGTATGTCATTAGCAGCCAAAATGGAGGAGACTAACGTGCCTCAGTTGGTAGATTTACAGGTGGGAGAACCTAAGTTTGTGTTTGGAGCTAACTCTATACAGAGAATGGAGCTTCTGGTGCTGGGCACATTGGGTTGGAGGATGCGTGCTCTTACCCCTTGCTCATTCATTGATTACTTTCTAAGCAAGATTAACAAAGTTCAGTATCCATCAATATTATCCATTTCTAGATCAGTACAACTCATATTAAGCACAATCAAAGGTATTGATTTCTTGGAATTCAGGCCCTCTGAAATTGCTGCAGCGGTGGCCATTTCTATTTCAAGGCAAATTCAAGAAGTGGACATTGATAAGGCCATGTCTTGTTTCAAACATGTAGAAAAGGAAAGGGTCGTGAAGTGTCTTGAACTGATGAAAGATTTGTCATTGATTAGTGGGTCTGCTAATTTGGCAAGTAACTTAGCTTCATCTGTGCCCCAAAGCCCTGTTGGGGTTTTAGATGCTGCTTGCTTGAGCTATAAAAGTGATGAATTAACAGTTGGGTCATGTGCAAATTCTTCACATAATAGTCCAGATACAAGGGGAAGACACCAGACAGATCATCTAAAGTAGAATTCTAGTCATGACGTGTGAATTTTTATCCTTCACACCCTAGTCCAATCAGTTTGGTTGAATGGGATTTTTGGTGTGGTTTTGGAAGTGC

>Quercus_contig_11685

GGACTTCGAGCATTGACACCACGTGTACAATCTGGGACATCGAGCGTGAAGTCGTGGACACCCAACTCATCGCTCACGACAAAGAGGTGTACGATATTGCTTGGGGCGGTGTTGGGGTTTTCGCTTCTGTTTCGGCCGATGGGTCTGTTCGTGTTTTCGATCTTCGCGACAAGGAACACTCGACTATCATCTATGAAAGCTCTGAGCCCGACACGCCTTTGGTTCGACTAGGATGGAACAAGCAGGATCCTAGGTACATGGCGACTATTATCATGGACAGTGCTAAGGTTGTTGTGCTTGACATTCGCTTTCCTACGTTGCCTGTTGTGGAATTGCAGAGACACCAAGCTTCTGTCAATGCGATTGCTTGGGCTCCACATAGTTCTTGCCATATCTGTACGGCTGGGGATGATTCTC

>Quercus_contig_15358

GCCACACGAGCTCATTCAATGGGTCTCAGCATAGTCAGTCCTTGCAGATTGTGCCACTACAGAATAAAGGATCATTGAAGGTGTTGCTCTTACATGGGAATTTGGATATTTGGGTCTATAGTGCACATAACCTTCCAAATATGGACATGTTCCATAAAACTTTGGGGGATATGTTTACAAGATTACCAGGGAACGTGAGCAACAAAATTGAAGGCCATATTAATCATAAAATCACTAGTGATCCTTATGTTTCAATCTCAGTATCAAATGCTGTAATTGGGAGGACTTTTGTGATTAGCAATAGCGAAAACCCTGACTGGAAGCAACATTTTTATGTTCCTGTAGCGCATCAAGCTGCTGAAGTGCATTTTGTGGTTAAAGATAGTGATGTTTTGGGGTCACAGCTTATAGGAGTTGTGGCAATTCCAGTGGAACAGATATACTCAGGGGCAAAGGTGGAAGGAACCTACCCAATCCTGAATAATAATGGGAAGCCTTGTAAGCCAGGGGCTGCCTTGAAGCTTTCAATTCAGTACACCCCAATTGAGAAATTGAGCATTTATCATCATGGAGTGGGGGCAGGCCCTGATTACCATGGGGTTCCTGGAACATACTTTCCTCTCAGGAAAGGTGGAACGGTAACTCTTTATCAAGATGCCCATGTTCCAGATGGGTCCCTTCCAAATGTGAAGCTTGATCAAGGGATGGATTATGTGCATGGGAAGTGTTGGCGTGACATTTTTGATGCCATACGTCTGGCCAGTCGTTTGATTTACATTACAGGGTGGTCAGTGTGGCACAAAGTTAGGTTGGTTCGGGATGCTGGTGATGCTTCAAATTACACCCTAGGAGACCTTCTGAAAACCAAGTCTCAAGAAGGAGTGAGAGTGCTACTTCTTATTTGGGATGACCCTACATCAAGAAACATTTTGGGTTACCAAACAGATGGAATCATGCAAACCCATGATGAGGAAACTCGCCGTTTCTTCAAACGCTCTTCTGTTCAAGTGCTACTTTGTCCCCGCATTGCAGGAAAGAGACATAGCTGGGTCAAGCAAAAGGAAGTCGAAACAATCTATACACACCATCAAAAAACTGTAATTGTGGATGCCGATGCTGGAAATTATAGAAGAAAAATCATCGCTTTTGTTGGAGGACTTGATTTATGTGATGGGCGATATGATACTCCACAGCACCCTATATTTAGGACACTACAAACAGTCCACAAGGATGACTATCACAATCCTACTTACGCGGGTAACACTGCTGGTTGTCCACGAGAGCCATGGCATGACTTGCACTCTAGAATTGATGGTCCAGCGGCATATGATGTCCTGACTAACTTTGAGGACCGCTGGCTTAAGGCTGCAAAACCCCATGGGATTCAAAAACTGAAAATGTCATATGATGATGCGTTGCTCAGGCTAGAAAGAATTCCGGAGATTATTGGACTCTCAGATGCTCCTTGGACTAATGATAATGATCCAGAAGCTTGGCATGTTCAAATTTTTCGTTCTATTGATTCAAATTCTGTTAAAGGGTTCCCTAAGGATCCAAGAGATGCCACAAGCAAGAACCTGGTGTGTGGGAAGAACTTGCTGATTGACATGAGCATACATACGGCATATGTAAAGGCCATTCGTGCTGCCCAGCATTTCATTTATATAGAGAACCAGTATTTCATTGGATCTTCATACAATTGGAGTTCACATAAAGACATAGGTGCTAATAATTTGATTCCAATGGAAATTGCCCTTAAGATTGCTGAGAAAATTAGAGCAAATGAGAGATTTGCTGTATATATTGTTATCCCAATGTGGCCAGAAGGTGCTCCAACTGGTAACGCTACTCAGAGGATTCTATATTGGCAGAATAAAACAATGCAAATGATGTATGAGACCATCTACAAGGCTTTGGTGGAGGTTGGGCTTGAGGAAGCATACTCACCACAAGATTATCTGAACTTCTTCTGCCTCGGCAACCGTGAAGCCATAGATGGATATGACACTACAGTGCCTGGAAGTGCTACTCCAGCTAACACTCCTCAGGCACAAAGTCGAAAAAACCGACGATTTATGATTTATGTTCATTCAAAGGGCATGATAGTTGATGACGAATACATAATAGTGGGTTCTGCAAACATCAACCAGCGCTCCATGGAGGGCACGAGAGACACTGAGATTGCAATGGGAGCATATCAACCGAATCATACCTGGGCAAGAAAATATTCTGATCCGCATGGACAGATTTATGGATATAGGATGTCATTATGGGCAGAACATCTTGGATTTACTGAAGACTGCTTCAAACAACCAGAGTCTCTTGATTGTGTGAGAAGAGTAAGATCACTAGGAGAGATGAACTGGAAACAATTTGCAGCCAATGAGATCATAGAGATGACAGGCCACCTATTAAAATACCCAGTTGAAGTTGATCGAAAGGGCAAGGTGAGGCCCCTTCCTGGATGTGAAACTTTCCCAGATGTTGGAGGAAGTATAGTTGGTTCATTTCTTGCCATTCAGG

>Quercus_contig_21908

GCACTTCAACAGACAGACGATTTAGTGAAGGCAACCGCATCTGCTGATCCTGTGCTCTACTCCTTTTGAAAGGAGGCTCCGGTGATGATGAAATGCACGACGCTGCAGCCCATGAGTCATTGGAGCTATCAAAGCTAAAAGATGCGTCAATGATGCCATTGGGGCTGCTGGGTATGGACAGATGCTTGCGCTTGCGGCTTTGGTCGTGAATGGGGCCAAGGCCATTAGATAATTCCAGGATCAGCTTATAGCACTCATTGACTTTTTCCTCACTAATTTTAAGTACACTCATAAGCTGATTCTGATATTCCAGCGGATTATAAGGCTCTATATCCCTAATTACATGAAGCATCGTTGCAGTAGCTAATACAGATGGAAGGTGACTTGTTACCCTTGAATCGGCAATGACAGAGAGATGAAGGCGCTCACACCTCAACAGGAAATCCCAATGCAAGTGGTTCTTCAAACCAAGCCTCCTGATAATGTGATAAAAGAATGAAATTGGGGTCACCGGGTTCATCCTCCATTGAAGAGTGGACAGCACCAGAAGCTCCATTCTCTTAATAGTCTTTGCTTCAAATACATACTTGGATTCCTCCACTTGCAGGTCTAAAAGAAGTGGCACTTGGGTTTCCTCCACCTTAGCAGCCAGAGATAGACAGGCCACTGCAGCAAGTTGAGTCATCCAGGGCTTATCCCTCTTAAACGTGGGGCTTGAAGCAAACCTATCAAAGTAATTCACAGCAAGAACAGCAGTCAAAGCACAGAACCCATAGTGTGCCTTAACCCTCAAAATCCAATCCACAGCCTCTTTCCTAGCCACCATTAGAGACCCATCTGAGATCAGACTGCTCAAACACACATGGGTCTCTCCCTCCTTGGATTTTAGGGACACAAGTTCATCATCCTCCCAAACCAAGTCATTCTCTAGCAAAACCGAAGGGAAAAATGAGTGCTTTTTCACATTCTCATCACTCTCTTCTTCCCCAATACCATTCTCTTCCTCTAAACTCTCTTCCTCACAGAACAAAGCATCAATGACCGTTGCAGGACTTAGCAGCTGTTGGGTTTCCTCTTCTTGCAGA

>Quercus_contig_22955

AGGTTCTGTTTAGCTTCCTCCTTTTAGAATCTGGACTATTATCTGAAAAATTTGCACATGACCCATCACTTTTATAGCTCAAGCATGCGGCATCCAACACCCCAATTGGGCTTTGTGGTACAGATGGGACTGAAGCAGTGGCATCTTTAAAAGACACACCAATTAGTGATAAATCACTGATCAGATTAACACACTTAAGCACTCTCTCCTTTTCTACATGTTGAATGAGAACAGATGCTGCTGCCTCAATGTCTATTGTTTGGGTCTCTCCTGTAACAACTATTGCCACAGCTGCTGCAACCTCTGAAGCCCTGAATTCCAAGAATTCAATTCCTTTAATTGTGCCCAATATGAGTTCGATTGATCTTAAAATGGATGTACCAAGTGGGAATTGATCACCATTGATCTTGAAAAGGAAGTAGTCTATGAATGAGAAAGGGGTAACCGCTTGCATCCTCCACCTCAAAGTACTCAAAACAAGAAGTTCCATCCTTTGTATTGTTCTTGCTTCAAACACAAATTTTGCATCACCCACCTGCAAATCTAGAGATAGAGGAACTTCAGTCTCTTCCATTTTGGCAGCAAGAGATAAACATGCCACAGCCAACAATTGCATTGTCCAAGCTTTGTTCTTAGGAAATTCATAAGCAGAAAGGAACCGATCCAAGTAGCTTATAGCTAAATATGCACATAGAGGTCCAAAATTGAAATGGGCATGAACCTTTCCAATCCAATCAACAGCTTGTTTTCTAGCCCCCAAGTCCAAATCACCGCTTAGTAGTTTTTTCAAGTAATCACCACCAGGCAAATACTGGCATTCCTTTTCAACCATCAAAGCCAAACACTCATCACTCTGCAATGGCACCCCATCATTACCGCCGCCGCCGCCACCACCACCAAAGCCACGGTCTTGATTACGGCTCCGATGACCCAGATGATGCCACGTGGCCTCAAACTCCTCCACCACAACCCCATAATCGTTATCATCTAAAATACTATTGTCCTCCCCACAGAGAAGGCTTGAAACCGCGTAGTCAA

>Quercus_contig_25616

GTCAGGCAAAGCAGAACCTCTCCCCATGACATAATCATCTAGATGAGCCTTCCATAGGCCTTGCATCATATCTTTCCCACTAAAACCTTGTGAAGTGAACTCTGCAAAAGCTCTTCTGCTTAAGAAAAGGGTTTTGTACAAGCCCTTAAAAGCTCGCATTGCTGCAACCATCTTATCCATATTTCCAAAATATGTAGCCACATAAGAATCACTATTAATAGACACATAATAATCCAATGCAGCTTTTGTGTTCCCGTGCATACTTGTGAAATCCTCATCACTTAGGAGGCTAGACTTGGTAACTACATTGGTGTAGACAGATGTTAAGCCTTCAATTTCCATCAGACCATCCCCAGCTGCCAAATATATATTTGTGTCTGTTGGAATGGAAAGTGATTGGAGTATGAAGGCCGTCTCATTAGGGGTAAGAGGACATTTTCCCCGTTTTCTCCACACGTGGGCCAACTCTCCAGTCCATGGTTTTCTATCTCCTCGTGCTGCTTCAATGGCTTTGACAGAAGATGGAGAAAGACCTGAGTATTCACATTGGCTGTATGCTACCATGTCAGGTTCAAATCGAAGGTGAAGTGAGAGAAAGGGTTTGGGTATGGCTTTTAAGAGCTCAGAAGCCTTGTTCTCCAAGGATCTAGTGAGGCGCAACGCACTGTAACAAGCTTGACAAAGGGAAGCTTTTGCATACAGAGGATACCTATCTCTTCTTTGGCTCATTGCTGGTGTAATTGAAATATAATGATGTTCCAGCAAGGATGGAAGAACACTTTCAATGTAATCAAATTGGACTTTGCGTTTGCTACAGTCTACTCGAAAAGGTTCTTTTGATGCAATTTCTGGTGGCAACTCTTTGACAACCTTAATAAACCCATTCATCTGTTGAATAAAGTATTCAACATCAAATACATCTGCAAAACCACTTGATTCATTCCAATATGCAGCCACTTCAAACTTTGGCAAAACCAAGGTTGCATTTAACAAACGGGCAATACCAACACCATCACATAAATCTCTTCGCATCTGATTGAGCCCACCGTAGCAATCAACTCGGATATATCCATTATTCTCAAGTGGCAAAGCTGCAGCATTTCAACAAAAAACCAATGCAATTGTCACTTAGGCGTCCTTGTAGCCACCACTTGCAA

>Quercus_contig_32077

CTGTGAGAAGCATTCAGTGGAGCTACGCAATCTTCTGGTCCATTTCAGCTAGACAACCAGGGGTCTTGAAGTGGGGTGATGGATACTACAACGGAGATATTAAGACAAGAAAAACAATTCAAACCATGGAACTTAATTCTGACCAACTGGGTTTGCAGAGGAGTGAGCAATTGAGAGAACTTTATGAGTCCCTCTCAGCTGGTGAAG

>Quercus_contig_35143

GAAAAGGGCCTTGGACAGTGGAGGAGGACAGAATCTTAATGGATCACATAAGGGTGCATGGAAAAGGGAAGTGGAACCGCATAGCTAAAACGACAGGTTTGAAGAGGTGTGGCAAAAGTTGCAGATTAAGATGGATTAACTATCTAAGTCCTGGTGTAAAGCGAGGTGATTTCTCTGAGGAAGAGGAAGACCTCATTATTCGGCTTCACAACCTCCTTGGAAACAGGTGGTCTTTGATTGCTGGGCGAGTACCTGGGAGAACTGACAATCAAGTTAAGAACCATTGGAACACTCATTTGTGCAAGAAGCTTGGGATCAAGAAAGGGAAAACC

>Quercus_contig_39550

GTTGCGGTTCACAGTGTTCAAACAACAGGTGTTGGGGGTGGTGATGTTCAGGAAGACCCTGAGCTTTCTTTGCTGGCAAAGAAAACTTTACGGAGCAAATCTAACTCCTTTGGTTCATACTTGCTCCGTAACACTTCCTCTCATATACGCCAGGTTTCTCAGGAGCTCAAACGTTTCACATCTCTTACAAGAAGACCTTCTGCTTCGACACGTTTTGATCGAACAAAGTCTGCCGCCGCTCATGCTTTGAAGGGTCTCAAGTTCATCGCCGCCAAGACAAGTGGCGCTGCTGGTTGGCCGGCCATCGAGAAGCGGTTCGATGATCTCACCGCTTCTAAAAATGGACTCCTTCCCTCGTCCTTGTTTGGAGAATGCATAGGAATGAACAAGGAGTCTAAGGAGTTCGCAGGTGAGATGTTCAGAGCACTCGCTCGGCGATATAACATTAGTGGTGATTCAATCAACAAGGCACAGCTGAAAGACTTCTGGGAACAAATCTCTGATGAAAGCTTCGACTCCAGGCTCCAAATTTTCTTTGACATGGTAGACAAAGATGCCGATGGAAGAATTGCAGAAGAAGAAGTCGCTGAGATTATCAGCCTTAGTGCTTCTACAAACAAACTTTCCAATATTCAGAAACAAGCCAAGGAATATGCAGCTTTAATTATGGAAGAACTAGACCCAGAAAATAATGGGTACATCATGATATACCATCTTGAAACACTGCTATTGCAAGCTCCAAACCAATCTGTCAGAATAAGTGATAGCCGAATTATGAGTCAAATGTTGAGTCAGAAACTGAAGCCAACACAGGAGGACAACCCAGTGAGAAGAGGGTACCAAAAGATTAAGTACTTTCTAATGGATAACTGGCAAAGGGTCTGGGTAATGATGTTATGGCTTGGGATCATGCTGGGTCTTTTTGCCTACAAATTTGTGCAGTATCGAAACAAGGCCGCATTCGAAGTGATGGGCTACTGTGTTTGCGTTGCAAAAGGAGGGGCAGAGACCCTTAAATTCAACATGGCTCTAATACTATTACCTGTCTGCCGGAACACCATTACTTGGCTTAGAAACAAAACCAAATTAGGGACCGTTGTTCCCTTTGATGACAATCTGAATTTCCACAAGGTAATTGCGGTTGGAATTGGGGTCGGGGTTGGACTACACGCGATTTCTCATTTAGTATGTGATTTCCCAAGACTTCTTCACGCAACTGCGGAACAGTACGAGCCCATGGTATATTACTTTGGGGAACAACCTACTAGCTACTGGTGGTTTGTGAAAGGAGTTGAAGGGGTCACAGGTATAATAATTGTGATCTTGATGGCCATAGCATTCACATTAGCTACCCCTTGGTTTAGACGAAACAAGCTCAACCTACCAAAGCCCCTCAAGAAATTTACTGGATTCAATGCCTTTTGGTATTCCCACCATCTTTTTGTCATTGTCTATGCCCTCCTCATTGTCCACGGCATCAAACTCTATATCACAAAAAAGTGGTATCAGAAAACGACATGGATGTATTTGGCAGTTCCTGTGACCCTCTATGCATGTGAAAGGTTAATTAGAGCTTTTAGATCAAGCATCAAGCCTGTGAAGATTCTAAAGGTTGCTGTTTATCCAGGAAATGTGTTAGCATTGCACATGTCGAAGCCTCAAGGTTTTAGATACAAGAGTGGACAGTATATGTTTGTCAACTGTGCTGCTGTCTCAGCCTTCGAATGGCACCCATTTTCCATTACGTCATCACCAGGAGATGATTACCTAAGTGTGCACATTAGAACACTAGGTGACTGGACACGGCAGCTCAAAACTGTTTTCTCTGAGGTGTGTCAACCTCCAACTGCTGGGAAGAGCGGATTACTCAGAGCTGATTTCATGCAAGGAGGTAACAACCCCAGCTTTCCAAAGATACTGATAGACGGTCCATATGGAGCTCCAGCACAAGATTACAAAAAATACGATGTGGTGTTACTAGTTGGGCTTGGGATTGGGGCCACTCCAATGGTTAGCATTGTAAAAGACATTATCAATAACATAAAAAACAAAGATATGGAAGATCAAGATTCAACAACTGAGGCTGCTTTGGAAAGTGGGAGAGGTAGCTCTTCATCAAGTCACAATAAGAACAAGGGATTCAGGACAAGAAAAGCCTATTTCTATTGGGTCACAAGGGAACAAGGCTCATTCGAGTGGTTCAAAGGGATAATGAATGAAGTGGCTGAAATGGATGAGAAACAAATGATAGAATTGCATAACTATTGTACAAGTGTGTATGAAGAAGGAGATGCAAGATCGGCACTCATAGCAATGCTTCAATCACTACACCATGCCAAGAGTGGTTTGGATGTGGTGTCTGGCACAAGAGTCAAATCCCACTTCGCCAAACCCAATTGGCGACAAGTCTACAAGAAGATTGCTCTTCACCATCCAGAAACACGAGTCGGA

>Quercus_contig_41116

GGAAACAAGCCAAGACCAGGAGCTCCACGTCAGAAGAAGTGAGCAGTATTGAGTGGGATTTCATAAAGATGACTGAACAAGAAGAGGATCTCATCTGTAGGATGTACAGACTTGTTGGAGACAGGTGGGATTTGATAGCAGGTCGGATTCCAGGGCGAAAACCAGAAGATATAGAGAGGTTTTGGATAATGAGACATGGTGAGGTATTTGC

>Quercus_contig_47871

CCCTAACACCTTCACTTGCCTTTTTCTTAAGCAGATCACCAAGTGAGATGTCTCCTCCAGACTTTGGCCTCCTTGAGTCCCTCACCAAGGAAATTTTTGTATAGACAGACCATCCGGTAATGTAGATCAAGTGCTTTGCATTAGTGATTGCATCAAAAACGTCTTCCCAACATCTGTGGGGCTCAT

>Quercus_contig_56291

GATGGAGGAAGGGAACACGTATAAGAAGGGTTTATGGACTAAGGAAGAAGACAAGATACTAACGGATTACATTAAGGTGCATGGAGAAGGAAAGTGGAATCTTGCTGCTAAGAAGACAGATTTGAAGAGATGTGGGAAGAGTTGTAGGTTAAGGTGGATGAATTACCTTAGTCCTAATGTGAAGCAAGGCAGTTTTACTGAGGAAGAAGAAGACCTCATTATCAGACTTCATAATCTCCTTGGAAACAGGTGGTCTTTGATTGCTAAACTGGTGCCTGGACGAACTGACAATCAAGTGAAAAACTACTGGAACTCTCATCTGAGCAAAAAGCTGGGCCTCAAGAAGCAAAA

>Quercus_contig_72031

CAGGAGATTCATTCAAGCAGCACAAGCTTCTTACAAGCTTCCTTCTATCGAACTTGAGCTCTTGGCAAATTATTTAGCAGAACTGACACTTCTCGAATATAGCTTCCTGAAGTTTCTACCTTCCCTCATTGCTGCAGCTGCTGTGTTCCTCGCCAGATGGACACTTAATCAGTCAGAACACCCATGGAATCTGACCTTAGAGCATTATACCAGTTACAATGCATCAGAACTGAAAACCACCGTCCTTG
