# Supplementary material for: RNA-Seq Analysis of Quercus pubescens Leaves: De Novo Transcriptome Assembly, Annotation and Functional Markers Development
Source: PLoS One. 2014 Nov 13;9(11):e112487. doi: 10.1371/journal.pone.0112487 (PMC4231058; doi:10.1371/journal.pone.0112487)
Supplement: Table S4 — Characteristics of the Q. pubescens microsatellites markers analyzed. (DOCX) [file pone.0112487.s006.docx]

**Table S4**.

| Locus name | Primer sequences 5’-3’ | Repeated motif | Allele size range (bp) | Total no. alleles |
| --- | --- | --- | --- | --- |
| Qp_ssr_130 | GTGGAGGAGTGAAGAGCGAG | (GT)_14_ | 252-254 | 2 |
|  | ACCCAACAACAACACCCAAT |  |  |  |
| Qp_ssr_184 | AGAAGCCCTTTGGCCTTTAG | (AG)_7_ | 298-306 | 4 |
|  | ATCCCTTGAGGGTTTGGTTT |  |  |  |
| Qp_ssr_279 | GGAGGCCACTAACTTGACCA | (CT)_7_ | 278-284 | 2 |
|  | ACCAAGACGATGAAGGATCG |  |  |  |
| Qp_ssr_410 | CGCAGTTGCTCTTGTCGTAT | (GA)_13_ | 244-246 | 2 |
|  | GAGGATCGGTGAGCAGAAAG |  |  |  |
| Qp_ssr_449 | TTGTTGGGATAGCAGAAGCTC | (CA)_13_ | 276-284 | 4 |
|  | AGACTTCTCCGGATGAGCAA |  |  |  |
| Qp_ssr_492 | CACACGCATTCATTTCAACC | (TC)_14_ | 198-204 | 4 |
|  | TTCTGGGATTCACAACACGA |  |  |  |
| Qp_ssr_553 | GGCCGTCCCTTATCTCCTTA | (AT)_9_ | 276 | 1 |
|  | GGGAAGAAACAAAGTCTTGCTG |  |  |  |
| Qp_ssr_623 | GGGAACGGAGCAAACATAGA | (GA)_8_ | 157 | 1 |
|  | TCAGCTCAAGCACCAATGAC |  |  |  |
| Qp_ssr_681 | CAACACACAACCTTCGTGCT | (TC)_13_ | 141-143 | 2 |
|  | CGAAGTTCCTGTAACAGAGGC |  |  |  |
| Qp_ssr_732 | AAAATATTGCAAGTGGGGCA | (TG)_8_ | 157 | 1 |
|  | ATCGCCACACACACATCAGT |  |  |  |
| Qp_ssr_768 | GCATTAGCTTGACGAGGAGG | (TC)_6_ | 297 | 1 |
|  | CTCTCTTTGTTTTGTCCCTCG |  |  |  |
| Qp_ssr_804 | AAATCGAGCCATTCACTGGT | (AG)_10_ | 255-261 | 3 |
|  | TGATAATTTTCTCCTCCTCCCA |  |  |  |
| Qp_ssr_31 | ACCACAGCAAAAACTGTCCC | (CAG)_7_ | 217 | 1 |
|  | GGGATCCAGGACTTGACAAA |  |  |  |
| Qp_ssr_102 | CTTTACAGCCAATCCCCAGA | (CGG)_6_ | 270 | 1 |
|  | CCAAAACCAGCACTGTGAGA |  |  |  |
| Qp_ssr_168 | GAAAGGTGGATTTGCTGGAA | (GAA)_9_ | 221-233 | 4 |
|  | CTATGAAGCGGGTCTGGAAA |  |  |  |
| Qp_ssr_298 | TCCAATACTTACCATCATCCACA | (ACA)_12_ | 217-223 | 3 |
|  | TCCCGATAATCCTCACATCC |  |  |  |
| Qp_ssr_430 | TAGAAATTGACTTGCCGACG | (ATT)_8_ | 266-272 | 2 |
|  | TTTTCTCTTTTGGTTTGGGG |  |  |  |
